# BCIW-Net: Boundary-Conditioned Adaptive CT Windowing for Hepatocellular Carcinoma Segmentation

**DOI:** 10.3390/cancers18203317

**Published:** 2026-10-14

**Authors:** Tripti Singh, Julia Rebecca Kampel, Farhan Akram, Yifan Li, Akshat Tiwari, Sheetal Santosh Deshpande, Shriya Bommaraveni, Mohamed Abdel-Nasser, Domenec Puig, Vivek Kumar Singh

**Affiliations:** 1 Department of Computer Engineering and Mathematics, Universitat Rovira i Virgili, 43007 Tarragona, Spain; tripti.singh@estudiants.urv.cat (T.S.); sheetalsantosh.deshpande@estudiants.urv.cat (S.S.D.); domenec.puig@urv.cat (D.P.); 2 Centre for Cancer Biomarkers & Biotherapeutics, Barts Cancer Institute, Queen Mary University of London, London EC1M 6BQ, UK; j.r.kampel@smd25.qmul.ac.uk (J.R.K.); qs260029@qmul.ac.uk (Y.L.); a.tiwari@smd26.qmul.ac.uk (A.T.); 3 Department of Pathology and Clinical Bioinformatics, Erasmus MC, 3015 GD Rotterdam, The Netherlands; f.akram@erasmusmc.nl; 4 Queen’s University Belfast, Belfast BT7 1NN, UK; sbommaraveni01@qub.ac.uk; 5 Electrical Engineering Department, Faculty of Engineering, Aswan University, Aswan 81528, Egypt; mohamed.abdelnasser@aswu.edu.eg

**Keywords:** hepatocellular carcinoma, computed tomography, tumour segmentation, adaptive intensity windowing, boundary-aware learning, medical image analysis

## Abstract

**Background/Objectives:** Hepatocellular carcinoma (HCC) is the most common primary liver malignancy, and accurate tumour segmentation in contrast-enhanced computed tomography is essential for treatment planning, assessment of ablation margins, and post-treatment evaluation. Existing deep learning methods typically use fixed-intensity CT preprocessing, overlooking the adaptive windowing employed by radiologists to enhance tumour visibility. To address this limitation, we propose BCIW-Net, a boundary-conditioned intensity-windowed network that integrates spatially adaptive and differentiable intensity windowing into the segmentation process as a clinically motivated inductive bias. **Methods:** The proposed framework first generates complementary soft-tissue and tumour-enhancing intensity representations using differentiable sigmoid-based transformations. A primary encoder–decoder module predicts tumour guidance, boundary probabilities, and pixel-wise error proxy maps, which collectively guide the dynamic estimation of spatially varying window centre and width parameters. The resulting adaptively windowed features are selectively blended with fixed-intensity representations through an error-gated mechanism before final refinement in a secondary encoder–decoder network. A multi-task objective jointly optimises tumour segmentation, boundary prediction, error-proxy estimation, and adaptive window regularisation in an end-to-end manner. **Results:** Experiments on the primary liver cancer (PLC)-CECT dataset demonstrate that BCIW-Net achieved a mean Dice similarity coefficient of 78.68% and an intersection over union (IoU) of 64.85% across five-fold patient-level cross-validation, outperforming the seven evaluated CNN-, Transformer-, and state-space baselines. Evaluation on LiTS2017 without dataset-specific fine-tuning yielded a tumour Dice score of 89.84%, providing preliminary evidence of cross-dataset generalisation. **Conclusions:** Qualitative feature visualisations show that adaptive windowing enhances tumour representation and boundary delineation, demonstrating the effectiveness and interpretability of BCIW-Net for HCC segmentation.

## Simple Summary

Hepatocellular carcinoma can be difficult to segment on CT because tumours vary in size, shape, enhancement, and boundary visibility. We developed BCIW-Net, a deep learning framework that adapts image intensity locally using tumour-related, boundary, and error information during segmentation. The method was evaluated on the PLC-CECT dataset using five-fold patient-level cross-validation and was also tested on LiTS2017 without dataset-specific fine-tuning. BCIW-Net improved tumour overlap and boundary localisation compared with the evaluated baseline models, suggesting that adaptive intensity transformation may help improve automated liver tumour segmentation.

## 1. Introduction

Hepatocellular carcinoma (HCC) is the most common primary liver malignancy and remains a major cause of cancer mortality worldwide. According to GLOBOCAN 2022 estimates, liver cancer accounted for approximately 866,000 new cases and 759,000 deaths globally, ranking sixth in cancer incidence and third in cancer-related mortality [1]. The aetiological distribution of HCC continues to change across regions. Chronic hepatitis B virus (HBV) and hepatitis C virus (HCV) infection remain major causes globally, although HBV vaccination, antiviral treatment for HBV, and direct-acting antiviral therapy for HCV are altering virus-associated disease patterns [2]. At the same time, metabolic dysfunction-associated steatotic liver disease (MASLD) and its inflammatory subtype, metabolic dysfunction-associated steatohepatitis (MASH), are increasingly relevant contributors to HCC risk, particularly in regions with a high prevalence of obesity and metabolic disease [2,3].

This growing disease burden highlights the critical need for accurate imaging for HCC diagnosis, staging, treatment planning, and post-treatment follow-up. Multiphasic contrast-enhanced computed tomography (CECT) is central to these clinical workflows. Images acquired during the arterial, portal venous, and delayed phases provide essential information regarding lesion enhancement patterns, forming the basis of the Liver Imaging Reporting and Data System (LI-RADS) [4,5]. Within this context, accurate tumour segmentation is essential for quantitative evaluations, including measuring total tumour burden, assessing treatment response via modified Response Evaluation Criteria in Solid Tumours (mRECIST) [6], planning thermal ablation and radiotherapy [7], and estimating future liver remnant volume before hepatic resection [8]. However, tumour boundaries remain predominantly manually delineated in routine clinical practice. This process is labour-intensive, time-consuming, and subject to considerable inter-observer variability, particularly for lesions with heterogeneous enhancement or poorly defined margins [9]. Automated tumour segmentation can therefore improve the efficiency, reproducibility, and objectivity of quantitative HCC assessment.

**Figure 1:**
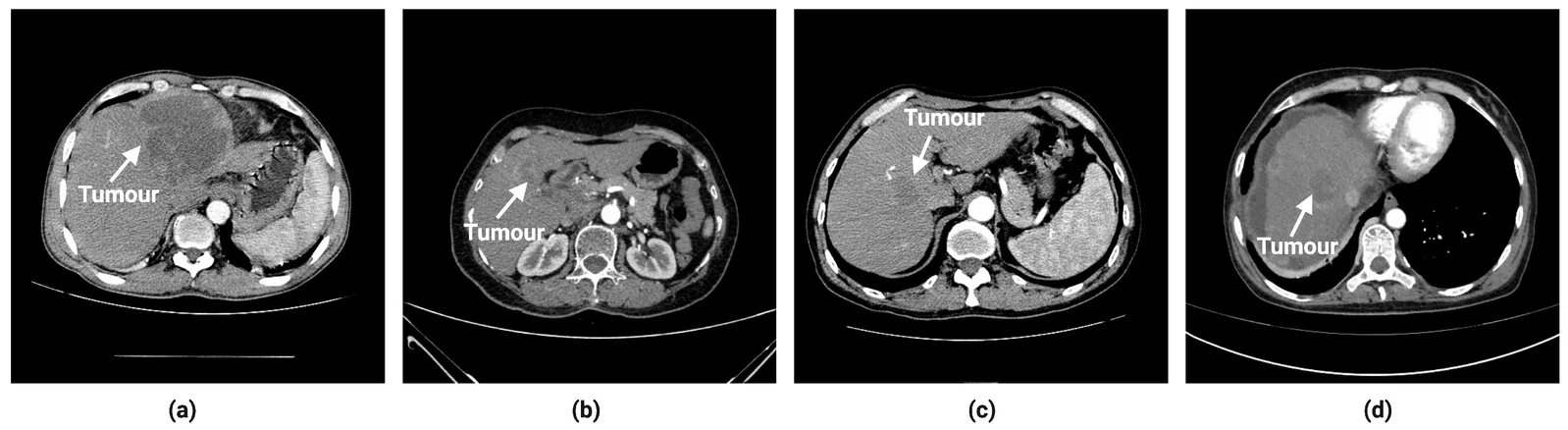
Representative contrast-enhanced computed tomography (CE-CT) images demonstrating the structural and enhancement heterogeneity of HCC tumours (indicated by white arrows). The panels illustrate marked variability across key clinical factors: (**a**) a large tumour, (**b**) a small tumour, (**c**) a centrally located tumour, and (**d**) a peripheral tumour. Such heterogeneity underscores the visual complexity that challenges automated tumour segmentation algorithms.

Despite these potential benefits, achieving reliable automated HCC segmentation remains challenging due to substantial inter-patient variability in CT image appearance. As shown in Figure 1, tumours differ markedly in size, shape, enhancement patterns, and location within the liver. This variability, combined with the low contrast between tumours and surrounding liver parenchyma, frequently obscures lesion boundaries and limits the robustness and generalisability of automated segmentation methods.

To address these limitations, deep learning methods have gained widespread use in medical image segmentation. Convolutional neural networks (CNNs) based on encoder–decoder architectures, particularly U-Net and its variants, have achieved strong performance by combining hierarchical feature extraction with multi-scale feature fusion through skip connections [10,11,12,13,14,15]. More recently, Transformer-based architectures, state-space models, and hybrid CNN–Transformer networks have improved long-range context modelling and the representation of complex anatomical structures, achieving state-of-the-art results in medical image segmentation tasks. Despite these advances, most existing methods rely on static, fixed-intensity CT preprocessing. Consequently, intensity normalisation fails to adapt to local image characteristics, variations in tumour appearance, or spatial differences in segmentation difficulty.

The limitations of fixed-intensity preprocessing are particularly pronounced in HCC segmentation, where heterogeneous arterial enhancement and weak tumour–parenchyma contrast frequently obscure lesion boundaries. Specifically, the intensity range required to enhance a lesion’s interior may differ from that needed to delineate its boundaries, making standard global intensity transformations suboptimal. In routine clinical practice, radiologists address this challenge by dynamically adjusting CT window settings during image interpretation. In contrast, deep learning models typically employ a fixed-intensity transformation during both training and inference, restricting the image representation from adapting to local anatomical and pathological variations. This gap between clinical workflow and standard computational methods motivates the development of a segmentation framework in which CT intensity windowing is learned as a spatially adaptive, task-conditioned operation rather than treated as a fixed preprocessing step.

**Figure 2:**
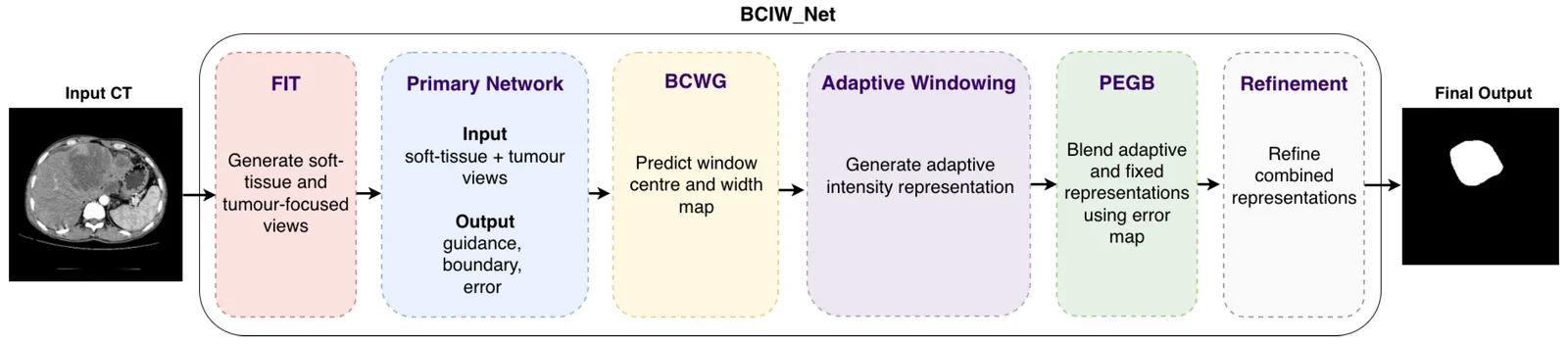
Overview of the BCIW-Net training pipeline. The input CT image is first transformed into complementary fixed-intensity representations, which are processed by the primary network to generate guidance, boundary, and error information. The Boundary-Conditioned Window Generator predicts spatially varying window parameters for adaptive intensity windowing, followed by predicted-error-gated blending and refinement to produce the final tumour segmentation mask. Training is jointly supervised using segmentation, boundary, error-prediction, and window-regularisation losses.

To address these challenges, we propose the Boundary-Conditioned Intensity-Windowed Network (BCIW-Net), a two-stage residual encoder–decoder framework that incorporates a differentiable, spatially adaptive CT intensity transformation directly into the segmentation process (Figure 2). In the first stage, the network generates complementary latent guidance, tumour boundaries, and error prediction maps. A Boundary-Conditioned Window Generator (BCWG) leverages these outputs to estimate spatially varying maps of the window’s centre and width. These parameter maps define an adaptive, sigmoid-based intensity transformation that selectively enhances local contrast based on predicted tumour characteristics. This enhanced representation is then combined with complementary fixed-intensity representations via a predicted-error-gated blending mechanism. Finally, a second encoder–decoder refines the combined representation to produce the final tumour segmentation. By embedding this adaptive intensity transformation directly into an end-to-end trainable framework, BCIW-Net allows feature representation to adapt to local tumour appearance, boundary characteristics, and predicted segmentation difficulty, ultimately improving the separation of challenging tumour regions from surrounding tissue. The contribution of BCIW-Net is not learnable CT windowing in isolation, but a spatially varying intensity-adaptation mechanism conditioned jointly on semantic features, predicted tumour boundaries, and a pixel-wise segmentation-error proxy. These signals are used to estimate local window-centre and window-width parameters, while predicted-error-gated blending controls the contribution of the adaptive representation before final segmentation refinement.

The main contributions of this work are summarised as follows:

1. We propose a spatially varying, boundary- and error-conditioned intensity-adaptation mechanism that jointly estimates local CT intensity transformation parameters with tumour segmentation.
2. We develop a Boundary-Conditioned Window Generator (BCWG) that combines decoder features, latent tumour guidance, predicted tumour boundaries, and error-proxy information to estimate spatially varying window-centre and window-width maps.
3. We introduce a predicted-error-gated adaptive blending (PEGB) mechanism that selectively combines adaptive and fixed-intensity representations, preserving global anatomical structures while enhancing local contrast in regions predicted to be difficult to segment.
4. We formulate a unified multi-task learning framework that simultaneously optimises tumour segmentation, boundary prediction, error minimisation, and adaptive window parameter regularisation through a single joint objective function.
5. We evaluate the proposed framework on the Primary Liver Cancer Contrast-Enhanced CT (PLC-CECT) dataset [16] and the Liver Tumour Segmentation (LiTS) 2017 benchmark [17]. Quantitative, qualitative, and ablation analyses demonstrate consistent performance gains over representative CNN-based, Transformer-based, and state-space segmentation methods.

## 2. Related Work

Artificial intelligence in medicine has recently expanded to foundation models and large language models (LLMs), with applications in clinical question answering, report generation, medical education, and decision support [18,19]. Multimodal models further integrate text with medical images, although challenges remain regarding factual reliability, bias, privacy, safety, and clinical validation [12,19,20]. The present study focuses specifically on pixel-level CT tumour segmentation, where accurate spatial representation and boundary localisation remain central.

### 2.1. Deep Learning-Based Liver and Tumour Segmentation

Automatic segmentation of the liver and hepatic tumours from CT images has been studied extensively because of its importance in tumour-burden quantification, treatment planning, surgical navigation, and longitudinal assessment. Despite considerable progress, hepatic tumour segmentation remains exceptionally challenging because tumours vary substantially in size, shape, attenuation, enhancement pattern, and anatomical location. Tumour boundaries are often indistinct because of heterogeneous enhancement, partial-volume effects, and minimal attenuation contrast between lesions and the surrounding liver parenchyma. The LiTS benchmark provides a widely used multi-institutional CT dataset and has demonstrated that accurate tumour segmentation is considerably more difficult than liver segmentation, particularly for small and low-contrast lesions [17].

To address these challenges, early deep learning methods commonly used a cascaded approach for liver and tumour segmentation. Christ et al. employed cascaded fully convolutional neural networks, in which the liver was first localised and a second network subsequently segmented the tumours within the predicted liver region [21]. By restricting tumour segmentation to the liver region, this approach reduced interference from unrelated abdominal structures and established a common strategy for hepatic tumour segmentation. However, liver-localisation errors inevitably propagated to the tumour-segmentation stage. Moreover, fixed image preprocessing limited the ability of the model to adapt to local variations in tumour appearance.

Following these early cascaded methods, U-Net-based encoder–decoder architectures became the dominant framework for liver and tumour segmentation because their skip connections integrate high-level semantic information with fine spatial details. Several studies extended U-Net to improve multi-scale feature extraction and tumour delineation. Seo et al. proposed a modified U-Net (mU-Net) that incorporated object-dependent high-level features into the skip pathways to improve the segmentation of small structures and liver tumours [22]. Residual and densely connected architectures have also been introduced to improve gradient propagation and feature reuse. For example, H-DenseUNet combined a two-dimensional (2D) DenseUNet with a three-dimensional (3D) counterpart, enabling the joint learning of intra-slice features and inter-slice volumetric context while reducing the computational cost of a fully 3D architecture [23]. In a broader approach to model configuration, nnU-Net demonstrated that dataset-specific adaptation of preprocessing, architecture, training, and post-processing can achieve strong segmentation performance without extensive manual architecture design [24]. However, while these methods substantially improved spatial feature extraction and optimisation, they fundamentally treat input intensity representations as static, unmodifiable inputs during inference.

### 2.2. Volumetric, Hybrid, and Multi-Scale Context Modelling

Liver tumours are 3D structures, and their appearance across adjacent CT slices provides important spatial context for segmentation. Purely 2D networks are computationally efficient and can be trained using large numbers of axial slices, but they do not explicitly model inter-slice continuity. In contrast, 3D networks capture volumetric relationships but require considerably more memory and computational resources. They may also be difficult to train when only limited data are available. To balance these advantages and limitations, hybrid architectures combine 2D feature extraction with 3D contextual modelling. For example, H-DenseUNet jointly learned 2D intra-slice features and 3D volumetric context through hybrid feature fusion [23], and was evaluated on the MICCAI 2017 Liver Tumour Segmentation Challenge and 3DIRCADb dataset.

In addition to volumetric modelling, multi-scale processing has been widely used to address variations in tumour size and morphology. Multi-scale feature aggregation, pyramid representations, dilated convolutions, and hierarchical decoding enable networks to combine local boundary details with broader anatomical context. TD-Net used a hybrid end-to-end architecture for automatic liver and tumour segmentation, combining complementary feature-processing pathways to improve tumour representation across multiple scales [25]. Similarly, recent multi-path architectures combine selective feature fusion with dedicated edge-processing branches to segment lesions of different sizes and those with poorly defined margins [26]. Despite these sophisticated spatial representations, these framework paradigms remain constrained by fixed CT intensity windows established during preprocessing, leaving them incapable of dynamically adapting contrast to resolve subtle local intensity variations.

### 2.3. Attention and Boundary-Aware Segmentation

Attention mechanisms have been incorporated into liver and tumour segmentation networks to suppress irrelevant features from surrounding abdominal structures and emphasise tumour-related information. RA-UNet combined residual learning with 3D attention modules to adaptively refine feature responses during liver and tumour segmentation [27]. More recently, MANet used multiple attention mechanisms to integrate contextual information and improve automatic liver and tumour segmentation in CT images [28]. Although these methods improve the selection of relevant features, their attention mechanisms are applied downstream, after the input images have undergone fixed-intensity preprocessing. Consequently, their ability to recover subtle diagnostic contrast that has been suppressed by initial intensity preprocessing may be limited.

In addition to selecting relevant features, accurate segmentation requires effective delineation of tumour boundaries. This requirement has motivated the development of boundary-aware learning methods. Edge-enhanced approaches use auxiliary contour targets, edge-prediction branches, or boundary-guided feature fusion to preserve fine structural information. Shui et al. proposed a three-path architecture that combined multi-scale selective feature fusion with edge-inspiring and edge-guiding branches to segment tumours of different sizes and those with poorly defined boundaries [26]. Similarly, MAEG-Net combined multi-scale adaptive feature fusion with edge-guided supervision to improve boundary detection in liver and tumour CT segmentation [29]. While these strategies underscore the necessity of explicit boundary constraints, they treat edge information merely as a passive auxiliary supervision signal or feature-level loss penalty, rather than utilising it to actively re-parameterise the underlying image intensity dynamic range.

### 2.4. Transformer-Based Liver Tumour Segmentation

Transformer-based segmentation architectures have recently emerged to address the limited receptive field of conventional convolutional networks. TransUNet combined a convolutional feature extractor with a Transformer encoder to capture long-range dependencies while preserving high-resolution features through skip connections for accurate localisation [30]. This approach catalysed the development of several Transformer-based and hybrid architectures designed specifically for liver and tumour segmentation. For example, DHT-Net introduced dynamic hierarchical Transformer modelling with an edge aggregation block for liver and tumour segmentation, using hierarchical representations to integrate local and global information [31]. CAFCT-Net combined convolutional and Transformer components with contextual and attention-based feature-fusion modules to improve liver and tumour segmentation [32]. Transformer architectures have also incorporated boundary information to improve tumour delineation. In another work, EG-UNETR integrated cross-level interactive Transformer modules with semantic enhancement and an edge-guided refinement block to improve contextual modelling and tumour-boundary delineation [33]. Although these methods improve the representation of long-range spatial relationships and multi-scale context, they incur heavy computational overhead and memory footprints. Moreover, they continue to rely on a fixed CT intensity representation defined a priori. Crucially, even global self-attention may have limited ability to recover low-contrast information that has been suppressed by initial intensity preprocessing.

### 2.5. Clinical Imaging Challenges, Intensity Representation, and Research Gap

The imaging appearance of hepatocellular carcinoma varies considerably across acquisition phases, lesion phenotypes, and treatment states, creating substantial challenges for automated tumour delineation. Arterial, portal venous, and delayed acquisitions may exhibit different tumour-to-liver contrast, while LI-RADS observations can vary in enhancement pattern, morphology, and margin conspicuity [5]. Post-treatment assessment introduces additional complexity because necrosis, perfusional alterations, residual enhancement, and treatment cavities may obscure viable tumour, with expected imaging appearances differing according to the type of locoregional therapy [34]. In some patients, previous allergic-like reactions or other clinical considerations may also limit the use of iodinated contrast media. These challenges differ further between non-contrast and contrast-enhanced CT. In non-contrast CT, the absence of enhancement information reduces lesion conspicuity and places greater reliance on subtle attenuation, texture, and structural differences. Recent work has therefore explored frequency-domain enhancement and cross-phase transfer learning to improve the representation of weak lesion boundaries in liver CT. For instance, S2DA-Net combines spatial and spectral feature learning to improve liver tumour segmentation, while other approaches use cross-phase information to enhance lesion conspicuity in non-contrast CT [35,36]. In contrast-enhanced CT, the availability of phase-dependent vascular information creates opportunities for adaptive intensity modelling and multiphase feature integration, while also introducing variability related to contrast timing and tumour vascularity. In Table 1, we summarise the challenges that motivate computational strategies which can accommodate heterogeneous lesion appearance, weak tumour–parenchyma contrast, and poorly defined boundaries. Such approaches are intended to support tumour delineation and should complement, rather than replace, established radiological assessment frameworks such as LI-RADS and treatment-response criteria. The present study evaluates BCIW-Net only on contrast-enhanced CT, and its performance on non-contrast CT requires separate validation.

From a computational perspective, most existing liver and tumour segmentation studies have focused on network architecture, loss design, feature fusion, and contextual modelling, whereas the role of CT intensity representation has received comparatively less attention. CT intensities are commonly clipped, normalised, or windowed before being provided to the segmentation network, and this transformation generally remains fixed across patients and spatial locations. Such preprocessing does not account for the local variation in lesion enhancement and boundary visibility described above. In clinical practice, radiologists adjust window centre and width settings according to tissue attenuation, enhancement phase, and diagnostic objective, suggesting that a fixed global intensity transformation may be suboptimal for heterogeneous HCC appearances. This limitation is particularly relevant when the intensity range that best represents the tumour interior differs from that required to delineate a weak or ill-defined boundary against the surrounding liver parenchyma. Attention mechanisms, boundary-aware objectives, and Transformer-based architectures can improve downstream feature representations, but they generally operate after the input intensity transformation has already been defined. Similarly, many boundary-guided methods use contour information as an auxiliary supervision signal or feature-level constraint without allowing it to influence the image intensity representation itself. To address this research gap, we propose BCIW-Net, which incorporates CT intensity transformation as a differentiable, spatially adaptive, and end-to-end trainable component of the segmentation framework. The model combines semantic guidance, predicted tumour boundaries, and pixel-wise error-proxy information to estimate spatially varying centre and width parameters for the proposed sigmoid-based transformation. The resulting adaptive intensity representation is combined with fixed-intensity representations through predicted-error-gated blending before final segmentation refinement. In this way, boundary and error information contribute directly to local intensity adaptation, enabling the input representation to respond to spatial variations in tumour appearance and segmentation difficulty.

## 3. Proposed Method

### 3.1. Overall Framework

**Figure 3:**
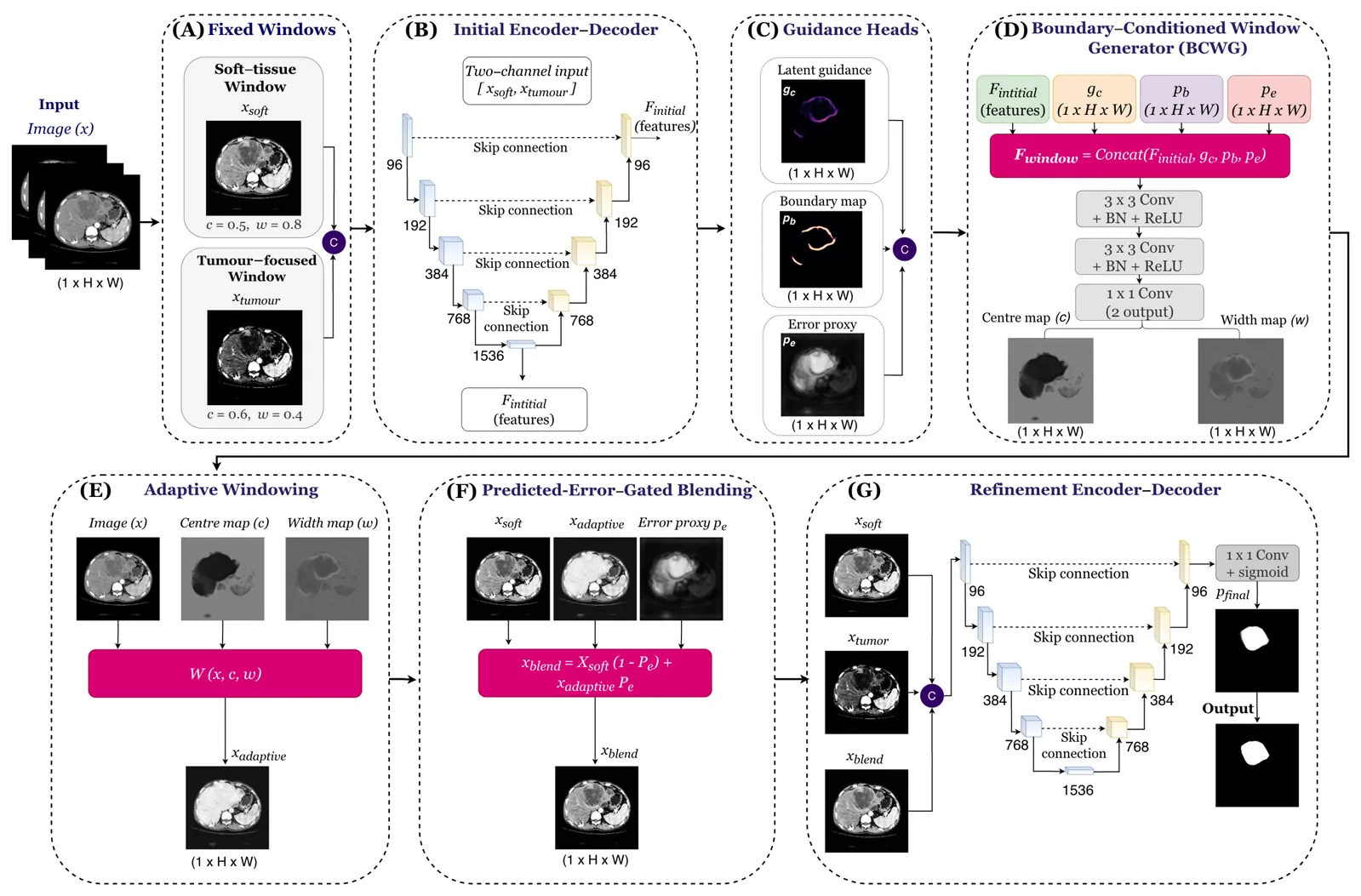
Detailed architecture of the proposed BCIW-Net for liver tumour segmentation. The framework comprises (**A**) fixed sigmoid-based intensity transformations that generate complementary soft-tissue and tumour-focused representations from the input CT image; (**B**) an initial residual encoder–decoder that extracts multi-scale features; (**C**) guidance heads that produce latent guidance, tumour-boundary probability, and error-proxy maps; (**D**) the BCWG, which combines the initial features and guidance maps to estimate spatially varying window-centre and window-width maps; (**E**) adaptive windowing, where the predicted parameters are applied to the input image using a differentiable sigmoid-based intensity transformation; (**F**) predicted-error-gated blending, which adaptively combines the fixed soft-tissue and adaptive intensity representations according to the error-proxy map; and (**G**) a refinement residual encoder–decoder that processes the complementary intensity representations to generate the final tumour segmentation. The overall framework enables boundary- and error-aware adaptation of local CT intensity contrast within an end-to-end trainable segmentation network.

An overview of the proposed BCIW-Net is presented in Figure 3. BCIW-Net consists of two sequential residual encoder–decoder stages connected by a BCWG, which estimates spatially varying intensity transformations for liver tumour segmentation. First, two complementary fixed-intensity representations are generated from the normalised input image using sigmoid-based window transformations. These representations are processed by the first residual encoder–decoder, which produces an intermediate feature representation and three complementary guidance outputs: a latent guidance map, a tumour boundary probability map, and an error prediction map. Instead of directly generating the final segmentation, the first stage provides semantic, structural, and error-aware information to guide the estimation of adaptive intensity windows. The BCWG then combines the first-stage feature representation with the three guidance maps to estimate spatially varying window centre and width maps. These parameter maps define an adaptive sigmoid-based intensity transformation that improves local image contrast according to the predicted tumour characteristics. The resulting adaptive intensity representation is combined with the two fixed-intensity representations using an error-gated adaptive blending mechanism. This process preserves global anatomical information while simultaneously enhancing locally discriminative tumour information. The blended intensity representation is subsequently processed by the second residual encoder–decoder to produce the final tumour segmentation. Through the joint optimisation of both stages, BCIW-Net learns the adaptive intensity transformation and tumour segmentation simultaneously in a unified end-to-end framework. The overall computational pipeline can be summarised as follows:

(1) $$x\to \left\{x_{\mathrm{soft}},x_{\mathrm{tumour}}\right\}\to F_{\mathrm{initial}}\to \left\{g_{\mathrm{guide}},p_{b},p_{e}\right\}\to \left\{c,w\right\}\to x_{\mathrm{adaptive}}\to x_{\mathrm{blend}}\to {\hat{p}}_{\mathrm{final}},$$

where *x* denotes the normalised input image; $x_{\mathrm{soft}}$ and $x_{\mathrm{tumour}}$ denote the fixed soft-tissue and tumour-focused intensity representations, respectively; $F_{\mathrm{initial}}$ denotes the first-stage feature representation; $g_{\mathrm{guide}}$, $p_{b}$, and $p_{e}$ denote the latent guidance activation, tumour boundary probability map, and error prediction map, respectively; *c* and *w* denote the spatially varying window-centre and window-width parameter maps; $x_{\mathrm{adaptive}}$ denotes the adaptive intensity representation; $x_{\mathrm{blend}}$ denotes the blended intensity representation; and ${\hat{p}}_{\mathrm{final}}$ denotes the final tumour segmentation probability map.

#### 3.1.1. Residual Convolutional Block

Both encoder–decoder stages are constructed using residual convolutional blocks to support effective feature learning and stable gradient propagation during optimisation. Given an input feature tensor *F*, each residual block contains two successive 3×3 convolutional layers, each followed by batch normalisation. A ReLU activation is applied after the first batch-normalisation layer. When the input and output channel dimensions differ, a 1×1 convolution is applied to the shortcut pathway to match their dimensions. The output of the residual block is defined as follows:

(2) $$X_{\mathrm{out}}=\mathrm{ReLU}\left(F(F)+S(F)\right),$$

where F(·) denotes the two-layer residual transformation, and S(·) represents either an identity mapping or a 1×1 convolutional projection, depending on whether the input and output share the same channel dimensions. Spatial downsampling is performed using a 2×2 maximum-pooling operation, followed by a residual convolutional block. During decoding, feature maps are upsampled using bilinear interpolation and subsequently passed through a 1×1 convolution to reduce the number of channels. The upsampled feature maps are subsequently concatenated with the corresponding encoder features using skip connections, and the combined features are processed by a residual convolutional block.

#### 3.1.2. Differentiable Sigmoid Intensity Transformation

BCIW-Net applies a differentiable sigmoid-based intensity transformation to the normalised input image. Let $x\in {\left[0,1\right]}^{H\times W}$ denote the normalised input image, and let $c,w\in R^{H\times W}$ represent the spatially varying window centre and width maps, respectively. For an input intensity x(i,j) at spatial location (i,j), the transformed intensity is defined as follows:

(3) $$W\left(x;c,w\right)\left(i,j\right)=\sigma \;\left(\frac{k\left[x(i,j)-c(i,j)\right]}{w(i,j)}\right).$$

Here,

$$\sigma \left(z\right)=\frac{1}{1+\exp(-z)}$$

denotes the sigmoid activation function, and k>0 is a fixed sharpness parameter that controls the steepness of the intensity transition. In all experiments, *k* was set to 8. The window centre c(i,j) determines the local transition point, whereas the window width w(i,j) controls the range over which the transition occurs. Larger values of w(i,j) produce broader and more gradual intensity transitions, whereas smaller values produce sharper transitions centred at c(i,j).

The proposed operator defines a monotonic, spatially varying, and fully differentiable intensity transformation that can be jointly optimised with the segmentation network through gradient-based learning. Although *c* and *w* are referred to as the window centre and window width, respectively, they parameterise the proposed sigmoid transformation rather than the conventional clipped-linear transformation used for CT windowing. Consequently, these parameters are not numerically equivalent to the CT window level and width used in standard radiological visualisation. Instead, they define a smooth approximation of CT windowing that supports stable end-to-end optimisation. To improve numerical stability and prevent excessively narrow intensity windows, the predicted window width is constrained to [0.05,1]. The lower bound prevents excessively steep transitions, whereas the upper bound limits the range of the predicted width and supports stable gradient propagation during network optimisation.

#### 3.1.3. Fixed-Intensity Representations

To provide complementary intensity information to the network, two fixed sigmoid-based intensity transformations are generated using the differentiable operator defined in Equation (3). The first uses a window centre of $c_{\mathrm{soft}}=0.5$ and a window width of $w_{\mathrm{soft}}=0.8$, producing a broad soft-tissue intensity representation. The second uses a window centre of $c_{\mathrm{tumour}}=0.6$ and a window width of $w_{\mathrm{tumour}}=0.4$, producing a tumour-focused representation that emphasises a narrower intensity range. The broader soft-tissue transformation produces a gradual transition that preserves a wide range of normalised image intensities and provides global anatomical context. In contrast, the tumour-focused transformation produces a steeper transition around its window centre, thereby improving local contrast within a more restricted intensity range. Together, these representations provide complementary anatomical and tumour-related intensity information for the subsequent adaptive-windowing process. The fixed window centre and width values were selected empirically during method development to produce complementary broad and tumour-focused representations, and were kept constant across all experiments. The resulting representations are concatenated along the channel dimension to form a two-channel tensor, which is provided to the initial encoder–decoder network. This concatenation retains the information contained in both intensity representations and allows the network to learn a joint feature representation.

#### 3.1.4. Initial Encoder–Decoder

The initial stage of BCIW-Net is implemented as a residual U-Net that processes the two fixed-intensity representations described above. The network uses a base channel width of C=96. Across five resolution levels, the encoder progressively increases the number of channels according to

{C,2C,4C,8C,16C}={96,192,384,768,1536}.

For an input image of 256×256 pixels, four successive downsampling operations reduce the feature-map resolution to 16×16 at the bottleneck while progressively increasing the number of channels. The decoder then restores the original spatial resolution through four upsampling stages. At each stage, skip connections transfer features from the corresponding encoder level to the decoder, combining fine spatial details with the high-level semantic information learned at deeper levels. The final decoder feature map is denoted by $F_{\mathrm{initial}}\in R^{C\times H\times W}$, where C=96, and *H* and *W* denote the height and width of the input image, respectively.

#### 3.1.5. First-Stage Guidance Heads

Three independent 1×1 convolutional heads are applied to the final decoder feature map $F_{\mathrm{initial}}$ to generate complementary guidance outputs for the adaptive intensity-windowing stage. The corresponding logits are defined as

(4) $$\begin{array}{cc} L_{\mathrm{guide}}\; & ={\mathrm{Conv}}_{1\times 1}^{\left(\mathrm{guide}\right)}\left(F_{\mathrm{initial}}\right), \end{array}$$

(5) $$\begin{array}{cc} L_{b}\; & ={\mathrm{Conv}}_{1\times 1}^{\left(b\right)}\left(F_{\mathrm{initial}}\right), \end{array}$$

(6) $$\begin{array}{cc} L_{e}\; & ={\mathrm{Conv}}_{1\times 1}^{\left(\mathrm{error}\right)}\left(F_{\mathrm{initial}}\right). \end{array}$$

The sigmoid function is then applied to obtain the corresponding activation maps:

(7) $$\begin{array}{cc} g_{\mathrm{guide}}\; & =\sigma (L_{\mathrm{guide}}), \end{array}$$

(8) $$\begin{array}{cc} p_{b}\; & =\sigma (L_{b}), \end{array}$$

(9) $$\begin{array}{cc} p_{e}\; & =\sigma (L_{e}). \end{array}$$

Although all three outputs use the same sigmoid activation, they serve different functions within BCIW-Net. The latent guidance activation $g_{\mathrm{guide}}$ provides high-level semantic information that conditions the BCWG during the estimation of spatially varying window centre and width maps. Unlike the final segmentation output, $g_{\mathrm{guide}}$ is not directly supervised and should therefore not be interpreted as a calibrated tumour probability map. Instead, it acts as an intermediate semantic representation that complements the structural and error-aware guidance signals.

The boundary probability map $p_{b}$ predicts tumour boundary locations and provides explicit structural information to guide the adaptive intensity transformation. The error prediction map $p_{e}$ estimates the pixel-wise segmentation error and is subsequently used as an adaptive gating signal during intensity blending. Together, the three outputs provide complementary semantic, structural, and error-aware information, allowing the BCWG to adapt the window parameters to local tumour characteristics and predicted segmentation difficulty.

#### 3.1.6. Boundary-Conditioned Window Generator

The BCWG estimates spatially varying window centre and width maps that define the adaptive sigmoid-based intensity transformation. Rather than relying only on image intensities, the BCWG conditions the transformation on complementary semantic, structural, and error-aware information produced by the initial encoder–decoder. The BCWG input is formed by concatenating the first-stage feature representation $F_{\mathrm{initial}}$, latent guidance activation $g_{\mathrm{guide}}$, boundary probability map $p_{b}$, and error prediction map $p_{e}$ along the channel dimension:

(10) $$F_{\mathrm{window}}=\mathrm{Concat}\left(F_{\mathrm{initial}},g_{\mathrm{guide}},p_{b},p_{e}\right),$$

where $F_{\mathrm{window}}\in R^{(C+3)\times H\times W}$, *C* is the number of channels in $F_{\mathrm{initial}}$, and *H* and *W* are the input image height and width, respectively.

The BCWG contains two successive 3×3 convolutional layers, each followed by batch normalisation and ReLU activation. The first convolution retains C+3 channels, whereas the second reduces the number of channels to

(11) $$C_{h}=\max\left(\left\lfloor \frac{C+3}{2}\right\rfloor ,8\right).$$

This reduction limits computational cost while retaining sufficient feature capacity. A final 1×1 convolution produces two output maps, $A_{c}$ and $A_{w}$, which are converted into the spatially varying window centre and width maps as follows:

(12) $$c\left(i,j\right)=\sigma \left(A_{c}\left(i,j\right)\right),$$

and

(13) $$w\left(i,j\right)=0.05+0.95\;\sigma \left(A_{w}\left(i,j\right)\right).$$

Thus, c(i,j)∈(0,1) and w(i,j)∈(0.05,1). The affine scaling of the predicted width restricts it to a stable range. In particular, the positive lower bound prevents excessively narrow transitions and supports stable gradient propagation during optimisation.

The predicted centre and width maps are then substituted into the differentiable sigmoid transformation defined in Equation (3) to generate the adaptive intensity representation:

(14) $$x_{\mathrm{adaptive}}=W\left(x;c,w\right),$$

where $x_{\mathrm{adaptive}}\in R^{H\times W}$. Unlike the fixed-intensity representations described previously, $x_{\mathrm{adaptive}}$ varies spatially according to the predicted semantic, boundary, and error-aware information. This adaptation enables the network to selectively enhance local image contrast in regions where tumour discrimination is predicted to be difficult.

#### 3.1.7. Predicted-Error-Gated Blending

The adaptive intensity representation is selectively combined with the fixed soft-tissue representation using the predicted error map $p_{e}$. Rather than replacing the fixed representation uniformly, $p_{e}$ controls the local contribution of the adaptive representation. This mechanism assigns greater importance to adaptive intensity enhancement in regions predicted to be difficult to segment. The blended intensity representation is computed through element-wise convex interpolation:

(15) $$x_{\mathrm{blend}}=x_{\mathrm{soft}}⊙\left(1-p_{e}\right)+x_{\mathrm{adaptive}}⊙p_{e},$$

where ⊙ denotes element-wise multiplication. When $p_{e}\left(i,j\right)$ is small, $x_{\mathrm{blend}}$ is dominated by the fixed soft-tissue representation, thereby preserving stable anatomical information. Conversely, as $p_{e}\left(i,j\right)$ increases, greater weight is assigned to the adaptive representation, increasing local contrast in regions predicted to require improved tumour discrimination. The proposed blending mechanism therefore introduces adaptive intensity enhancement primarily in challenging regions while limiting unnecessary changes to regions that are adequately represented by the fixed transformation. The resulting predicted-error-gated representation is then provided as input to the refinement encoder–decoder.

#### 3.1.8. Refinement Encoder–Decoder

The refinement stage uses the same residual encoder–decoder architecture and base channel width as the initial stage. Its input is formed by concatenating the fixed soft-tissue representation, fixed tumour-focused representation, and predicted-error-gated blended representation along the channel dimension:

(16) $$x_{\mathrm{refine}}=\mathrm{Concat}\left(x_{\mathrm{soft}},x_{\mathrm{tumour}},x_{\mathrm{blend}}\right),$$

where $x_{\mathrm{refine}}\in R^{3\times H\times W}$. The refinement network therefore processes three complementary intensity representations that provide broad anatomical context, enhance tumour-related intensity contrast, and introduce spatially adaptive intensity information, respectively. Unlike the BCWG, the refinement network does not directly receive the latent guidance activation $g_{\mathrm{guide}}$. Instead, the guidance information influences the refinement stage indirectly through the estimated adaptive window parameters and the resulting blended representation.

Let $F_{\mathrm{refine}}$ denote the final decoder feature map produced by the refinement network. The final segmentation logits are generated by a 1×1 convolutional prediction head:

(17) $$L_{\mathrm{final}}={\mathrm{Conv}}_{1\times 1}^{\left(\mathrm{final}\right)}\left(F_{\mathrm{refine}}\right).$$

A sigmoid activation is then applied to obtain the final tumour probability map:

(18) $${\hat{p}}_{\mathrm{final}}=\sigma \left(L_{\mathrm{final}}\right).$$

During inference, the final binary tumour mask is obtained by applying a decision threshold τ:

(19) $$\hat{y}\left(i,j\right)=1\left[{\hat{p}}_{\mathrm{final}}\left(i,j\right)>\tau \right],$$

where 1[·] is the indicator function. The threshold τ is selected exclusively from the validation set and then applied without modification to all test images.

### 3.2. Multi-Task Objective

BCIW-Net is trained using a multi-task objective with four complementary components: final segmentation loss, boundary prediction loss, error prediction loss, and adaptive window parameter regularisation. The segmentation loss directly supervises tumour delineation, while the auxiliary losses support boundary localisation, prediction of segmentation-error patterns, and stable estimation of adaptive window parameters. The latent guidance activation $g_{\mathrm{guide}}$ receives no direct supervision. Instead, it is learned implicitly through gradients propagated from the adaptive window generation and final segmentation objectives.

#### 3.2.1. Final Segmentation Loss

The final segmentation objective combines soft Dice loss with binary cross-entropy (BCE) computed directly from the final segmentation logits:

(20) $$L_{\mathrm{seg}}=L_{\mathrm{Dice}}\left(L_{\mathrm{final}},y\right)+L_{\mathrm{BCE}}\left(L_{\mathrm{final}},y\right),$$

where $L_{\mathrm{final}}$ denotes the final segmentation logits and *y* denotes the binary ground-truth mask. The soft Dice loss is defined as

(21) $$L_{\mathrm{Dice}}=1-\frac{1}{N}\sum_{n=1}^{N}\frac{2\sum_{i}p_{n,i}y_{n,i}+\epsilon }{\sum_{i}p_{n,i}+\sum_{i}y_{n,i}+\epsilon },$$

where $p_{n,i}=\sigma \left(L_{\mathrm{final},n,i}\right)$, *N* is the batch size, *i* indexes the spatial elements of sample *n*, and $\epsilon =10^{-7}$ ensures numerical stability.

#### 3.2.2. Boundary Loss

To provide explicit supervision of tumour contours, a morphological boundary target is generated from the binary ground-truth mask. Let ${\mathrm{MaxPool}}_{5}$ denote a 5×5 maximum-pooling operation with unit stride and padding of two pixels. The dilated and eroded masks are defined as

(22) $$y_{\mathrm{dilate}}={\mathrm{MaxPool}}_{5}\left(y\right),\;y_{\mathrm{erode}}=-{\mathrm{MaxPool}}_{5}\left(-y\right),$$

respectively. The boundary target is then calculated as

(23) $$y_{b}=\mathrm{clip}\left(y_{\mathrm{dilate}}-y_{\mathrm{erode}},0,1\right).$$

The boundary prediction loss is defined as

(24) $$L_{\mathrm{boundary}}=L_{\mathrm{Dice}}\left(L_{b},y_{b}\right)+L_{\mathrm{BCE}}\left(L_{b},y_{b}\right),$$

which encourages accurate localisation of tumour contours.

#### 3.2.3. Error Prediction Loss

The supervision target for the error prediction head is defined as the absolute pixel-wise difference between the final segmentation probability and the ground-truth mask:

(25) $$y_{e}=\left|\mathrm{stopgrad}\left({\hat{p}}_{\mathrm{final}}\right)-y\right|.$$

The stop-gradient operator prevents gradients from propagating through the final segmentation probability along the error-target construction pathway. The corresponding error prediction loss is

(26) $$L_{\mathrm{error}}=L_{\mathrm{BCE}}\left(L_{e},y_{e}\right),$$

where $L_{e}$ denotes the error prediction logits. Thus, the error head learns a proxy for the spatial distribution of segmentation errors rather than a probabilistic estimate of epistemic or aleatoric uncertainty.

#### 3.2.4. Adaptive Window Parameter Regularisation

The predicted window centre and width maps are weakly regularised around reference values of 0.5 and 0.4, respectively:

(27) $$L_{\mathrm{window}}=E_{i,j}\left[{\left(c(i,j)-0.5\right)}^{2}\right]+E_{i,j}\left[{\left(w(i,j)-0.4\right)}^{2}\right].$$

These reference values are derived from the parameter settings used for the fixed-intensity transformations. The regularisation discourages unstable or degenerate parameter estimates while allowing spatial variation when it improves segmentation performance.

#### 3.2.5. Total Loss

The overall training objective is defined as

(28) $$L_{\mathrm{total}}=L_{\mathrm{seg}}+\lambda_{b}L_{\mathrm{boundary}}+\lambda_{e}L_{\mathrm{error}}+\lambda_{w}L_{\mathrm{window}},$$

where $\lambda_{b}=0.3$, $\lambda_{e}=0.1$, and $\lambda_{w}=0.001$. These weights were kept fixed across all experiments.

### 3.3. Implementation Details

We trained all the models for 200 epochs using the AdamW optimiser with an initial learning rate of $5\times 10^{-5}$, a weight decay of $10^{-4}$, and a batch size of four. Cosine annealing was used to adjust the learning rate, and gradient clipping was applied with a maximum norm of five. Model selection was based exclusively on the validation Dice score. For each evaluated checkpoint, a single global decision threshold was selected from {0.30,0.35,…,0.70} to maximise the mean validation Dice score, using the same test-time augmentation protocol as that applied during inference. The checkpoint–threshold pair with the highest validation Dice score was retained, and the selected threshold was applied unchanged to all test images. No test data were used for model or threshold selection, and the test set was reserved exclusively for final evaluation. Training augmentation included horizontal and vertical flips with probabilities of 0.5 and 0.2, respectively, and random affine transformations with a probability of 0.7. The affine transformations included rotations of up to $\pm 25^{\circ }$, translations of up to ±5%, scaling factors between 0.90 and 1.10, and shearing of up to $\pm 5^{\circ }$. Photometric transformations were applied only to the input images and included brightness and contrast adjustments, gamma transformation, Gaussian noise, and 3×3 Gaussian blurring, with probabilities of 0.6, 0.4, 0.3, and 0.2, respectively. After photometric augmentation, the normalised image intensities were clipped to [0,1] to maintain the valid input range. All models were implemented in PyTorch 2.10.0 and trained using an NVIDIA GeForce RTX 4090 GPU with 24 GB of memory.

### 3.4. CT Preprocessing

The source CT images were clipped to an intensity range of −200 to 200 HU, corresponding to a conventional window level of 0 HU and a window width of 400 HU. The original intensity range before clipping was not reported in the source dataset publication and cannot be recovered from the available clipped images. Each 2D CT slice was subsequently normalised independently using min–max normalisation:

$$x_{\mathrm{norm}}=\frac{x-x_{\min}}{x_{\max}-x_{\min}}.$$

The resulting values were multiplied by 255 and converted to 8-bit grayscale PNG images. During model loading, the PNG values were converted to floating-point values and divided by 255, yielding network inputs in the range [0,1]. After intensity normalisation, images were resized from 512 × 512 to 256 × 256 pixels using bilinear interpolation. Corresponding tumour masks were binarised and resized using nearest-neighbour interpolation. Because normalisation is performed independently for each slice, a normalised intensity value does not retain a fixed correspondence to absolute HU values across slices. The adaptive window-centre and window-width parameters are therefore expressed in normalised-intensity units.

### 3.5. Datasets

The proposed framework was evaluated using two contrast-enhanced CT datasets for liver tumour segmentation: PLC-CECT [16] and LiTS2017 [17]. The PLC-CECT dataset comprises 278 patients with liver cancer and 83 non-cancer controls, with multiphasic CT acquisitions available in the plain, arterial, portal venous, and delayed phases. For this study, model development and evaluation were performed using arterial-phase images from 94 patients with pathologically confirmed HCC. Performance was evaluated using five-fold patient-level cross-validation. Patients, rather than individual image slices, were assigned to folds so that all arterial-phase slices from the same patient remained within a single fold. In each iteration, one fold was reserved as the test set, while the remaining patients were used for model development and were further divided at the patient level into training and validation subsets according to the predefined 70/10/20 training/validation/test proportion. The validation subset was used for checkpoint selection and decision-threshold optimisation, whereas the held-out test fold was used only for final performance evaluation. This process was repeated across all five folds, allowing each patient to contribute once to the held-out test evaluation. To evaluate the generalisability of the proposed framework, additional experiments were performed using the publicly available LiTS2017 dataset. This dataset consists of 131 3D abdominal CECT scans with expert annotations of the liver and tumours. It includes substantial variation in tumour size, morphology, and imaging appearance, making it a challenging benchmark for liver tumour segmentation. In the present study, LiTS2017 was used exclusively as an external evaluation cohort. BCIW-Net was trained only on the PLC-CECT dataset and was subsequently applied directly to all 131 publicly labelled LiTS2017 volumes. No LiTS2017 images were used for training, fine-tuning, validation, checkpoint selection, or decision-threshold optimisation. Although these 131 volumes correspond to the publicly released training cohort of the original LiTS challenge, they were not divided into study-specific training, validation, and test subsets in this work. For both datasets, tumour segmentation was formulated as a binary pixel-wise classification task. Tumour tissue was defined as the foreground class, while all other image regions were assigned to the background class.

### 3.6. Evaluation Metrics

We evaluated segmentation performance using the Dice similarity coefficient (Dice), intersection over union (IoU), precision, recall, and specificity. Let TP, TN, FP, and FN denote the numbers of true-positive, true-negative, false-positive, and false-negative pixels, respectively. The Dice and IoU scores for each test image were calculated as follows:

(29) $$\begin{array}{cc} \mathrm{Dice}\; & =\frac{2\mathrm{TP}}{2\mathrm{TP}+\mathrm{FP}+\mathrm{FN}}, \end{array}$$

(30) $$\begin{array}{cc} \mathrm{IoU}\; & =\frac{\mathrm{TP}}{\mathrm{TP}+\mathrm{FP}+\mathrm{FN}}. \end{array}$$

Precision, recall, specificity and Hausdorff distance (HD95) were calculated using their standard definitions. Following the evaluation recommendations of Taha and Hanbury [37], a Dice score and an IoU score of 1.0 were assigned when both the predicted segmentation and the ground-truth mask contained no tumour pixels, indicating correct identification of tumour absence. Conversely, when the ground-truth mask contained tumour pixels but the predicted segmentation was empty, Dice and IoU scores of 0.0 were assigned. All evaluation metrics were calculated independently for each test image and reported as means across the complete test set.

## 4. Results

### 4.1. Five-Fold Cross-Validation Performance

The segmentation performance of the proposed BCIW-Net was evaluated on the PLC-CECT dataset using five-fold patient-level cross-validation. Patient-level partitioning ensured that images originating from the same patient were confined to a single fold, thereby avoiding information leakage between the training and held-out subsets. The reported results therefore represent performance across independent patient groups rather than a single train–test partition. Table 2 reports the fold-wise performance in terms of Dice, IoU, precision, recall, specificity, and the HD95.

Across the five held-out folds, BCIW-Net achieved a mean Dice score of 78.68% ± 1.05 and a mean IoU of 64.85% ± 1.42. The corresponding mean precision and recall were 79.87% ± 1.07 and 77.52% ± 1.03, respectively. Specificity remained consistently high across the five folds, with an average value of 97.81% ± 0.06. The strongest fold-wise performance was observed in Fold 4, where the model attained a Dice score of 80.26%, an IoU of 67.03%, and an HD95 of 8.35 pixels. In contrast, Fold 3 yielded the lowest overlap performance, with a Dice score of 77.34% and an IoU of 63.05%, together with the highest HD95 of 10.31 pixels. This inverse tendency between overlap measures and boundary distance is consistent with the expected behaviour of these metrics: improved spatial overlap is generally accompanied by smaller discrepancies between the predicted and reference contours. The mean HD95 across the five folds was 9.19 ± 0.71 pixels, indicating relatively consistent boundary localisation across the held-out patient subsets.

### 4.2. Comparison with State-of-the-Art Segmentation Methods

Table 3 presents the quantitative segmentation performance of the proposed BCIW-Net alongside seven representative convolutional, Transformer-based, and state-space architectures on the PLC-CECT dataset. For BCIW-Net, the reported values correspond to performance aggregated across five patient-level cross-validation folds, whereas the comparison methods are reported using their corresponding evaluation results shown in the table. Among the convolutional architectures, U-Net [10] produced the lowest overlap performance, with a Dice score of 70.08% and an IoU of 53.94%. Attention U-Net [13] improved these values to 72.20% and 56.49%, respectively, while UNet++ [14] achieved a Dice score of 73.99% and an IoU of 58.71%. ResUNet [15] attained the strongest Dice performance among the convolutional baselines, reaching 75.51%, together with an IoU of 60.66%. EfficientUNet [38] achieved comparable performance, with a Dice score of 75.13% and an IoU of 60.17%. The Transformer-based Swin-UNet [39] further improved the segmentation results, attaining a Dice score of 76.71% and an IoU of 62.23%. Mamba-UNet [40] provided the strongest baseline performance overall, with a Dice score of 77.19%, an IoU of 62.85%, a precision of 78.23%, a recall of 76.17%, and a specificity of 97.56%. The proposed BCIW-Net achieved the highest performance across all reported overlap and classification metrics, obtaining a Dice score of 78.68%, an IoU of 64.85%, a precision of 79.87%, a recall of 77.52%, and a specificity of 97.81%. Relative to ResUNet, BCIW-Net improved the Dice and IoU by 3.17 and 4.19 percentage points, respectively. Compared with Mamba-UNet, the strongest overall baseline, BCIW-Net increased the Dice by 1.49 percentage points and the IoU by 2.00 percentage points. Precision and recall also increased by 1.64 and 1.35 percentage points, respectively. Boundary accuracy exhibited a similar ordering. BCIW-Net obtained the lowest HD95 value of 9.19 pixels, compared with 10.12 pixels for Mamba-UNet and 10.87 pixels for Swin-UNet. The lower HD95 indicates that the predicted lesion boundaries were, on average, closer to the reference contours. Taken together, the improvements in Dice, IoU, precision, recall, specificity, and HD95 indicate that BCIW-Net provided stronger region-overlap and boundary-localisation performance than the compared architectures on the PLC-CECT dataset.

### 4.3. Tumour-Positive and Boundary-Sensitive Evaluation

An additional evaluation was conducted using only slices containing tumour tissue in the reference annotation, and Table 4 shows whether the reported segmentation performance was influenced by tumour-negative slices. For tumour-positive-slice Dice, all reference-positive slices were retained, including cases in which the model produced an empty prediction; these cases were assigned a Dice score of zero. This evaluation removes the contribution of correctly predicted tumour-negative slices and therefore provides a more direct assessment of segmentation performance on tumour-containing images. Boundary agreement was additionally assessed using the HD95, computed between the predicted and reference tumour boundaries on the resized 256×256 masks. The HD95 is reported in pixels because the 2D CT slice image in PNG representation used for evaluation does not preserve the original voxel spacing and spatial geometry required for reliable conversion to millimetres.

The boundary-sensitive analysis showed a corresponding improvement in spatial localisation. BCIW-Net obtained a 2D HD95 of 9.87 pixels, compared with 11.42 pixels for Mamba-UNet. The lower HD95 indicates that the predicted tumour boundaries were generally closer to the reference contours. BCIW-Net also produced fewer complete tumour misses, with 14 missed slices among the 610 tumour-positive test slices, corresponding to a miss rate of 2.30%. In comparison, Mamba-UNet missed 21 tumour-positive slices, corresponding to a miss rate of 3.44%. A similar reduction was observed for false-positive detections. BCIW-Net produced 19 false-positive slices, whereas Mamba-UNet produced 26. Taken together, the tumour-positive Dice coefficient, HD95 value, missed-detection count, and false-positive count provide complementary evidence that BCIW-Net maintained its segmentation advantage when evaluation was restricted to clinically relevant tumour-containing slices and when boundary localisation was assessed directly.

### 4.4. Performance Distribution Analysis

**Figure 4:**
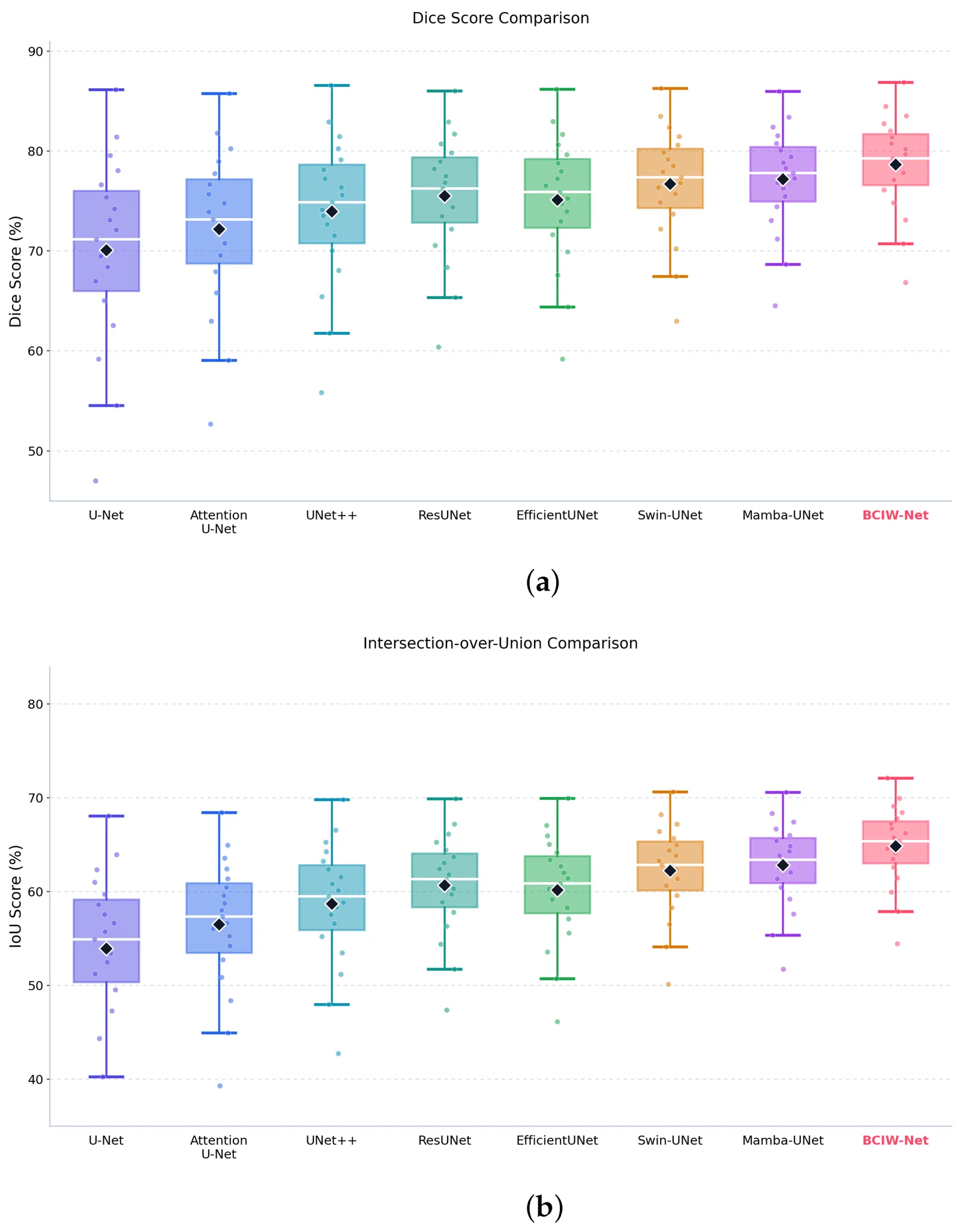
Box-plot comparison of Dice and IoU scores across eight segmentation methods. Each box represents the interquartile range (IQR), the white line indicates the median, and the diamond marker (⧫) indicates the mean. The whiskers extend to the most extreme values within 1.5× IQR of the lower and upper quartiles, while values outside this range are displayed as individual points. BCIW-Net, shown on the right and highlighted in red, achieves the highest median and mean for both the Dice and IoU. (**a**) Distribution of Dice scores on the test. (**b**) Distribution of IoU scores on the test.

Figure 4 illustrates the distributions of Dice and IoU scores for the evaluated segmentation methods on the independent PLC-CECT test set. While Table 3 reports the mean performance of each method, the box plots elucidate the variation in segmentation accuracy across individual test images. BCIW-Net achieved the highest mean Dice and IoU scores, reaching 78.68% and 64.85%, respectively, with its score distributions distinctly skewed towards higher values compared to competing architectures. Moreover, its relatively narrow interquartile ranges indicate more consistent segmentation performance across the test set. Several outliers were observed across all models, potentially reflecting variations in tumour size, morphology, location, and imaging appearance. Nevertheless, the higher median values and overall distributions obtained by BCIW-Net confirm that its gains were consistently distributed throughout the test cohort rather than driven by a minority of exceptional cases. Therefore, the box-plot analysis complements the mean results reported in Table 3, confirming that BCIW-Net achieves higher segmentation accuracy across diverse tumour appearances while maintaining relatively low performance variability.

To further evaluate the proposed framework on a public benchmark, Table 5 presents a quantitative comparison with recent liver tumour segmentation methods on the LiTS2017 dataset using the tumour Dice similarity coefficient. The hybrid architectures MA-cGAN, RHEU-Net, and ELANRes-MSCA-UNet reported tumour DSC values of 78.53%, 70.19%, and 72.90%, respectively, whereas the Transformer-based Improved Swin-UNet achieved a tumour DSC of 76.14%. Among the previously published methods included in the comparison, G-UNETR++ achieved the highest reported performance, with a tumour DSC of 84.40%, underscoring the potential benefits of integrating convolutional and Transformer-based feature representations. Under the evaluation protocol employed in this study, BCIW-Net achieved a tumour DSC of 89.84%, exceeding the value reported for G-UNETR++ by 5.44 percentage points. However, this cross-study comparison should be interpreted cautiously because the cited studies may differ in their data partitions, preprocessing procedures, model dimensionalities, post-processing techniques, and metric-aggregation strategies. Consequently, the results presented in Table 5 do not constitute a strictly controlled head-to-head comparison. Nevertheless, the LiTS2017 results provide additional evidence of the ability of BCIW-Net to segment liver tumours exhibiting considerable variations in morphology, size, and intensity distribution.

### 4.5. Ablation Study

#### 4.5.1. Effect of Individual Components of BCIW-Net

A cumulative ablation study was conducted to evaluate the incremental contributions of the main components of BCIW-Net. The evaluated components included fixed sigmoid-based intensity transformations (FITs), the two-stage encoder–decoder architecture (2S), the Boundary-Conditioned Window Generator (BCWG), predicted-error-gated blending (PEGB), boundary supervision $L_{\mathrm{boundary}}$, segmentation-error supervision $L_{\mathrm{error}}$, and adaptive-parameter regularisation $L_{\mathrm{window}}$. Because the components were introduced sequentially, the differences between consecutive variants represent conditional contributions under the evaluated order rather than independent effects.

As shown in Table 6, the cumulative ablation analysis shows progressive improvements as the individual components are incorporated into BCIW-Net. Starting from the single-stage raw-input baseline (V1), introducing FITs increased the Dice score from 70.26% to 72.62% and the IoU from 54.15% to 57.01%, while the HD95 decreased from 15.84 to 14.72 pixels. Addition of the two-stage architecture (V3) further improved the Dice and IoU to 73.91% and 58.62%, respectively, with a corresponding reduction in the HD95 to 13.61 pixels. Incorporating the BCWG increased the Dice score to 75.21% and the IoU to 60.27%, while precision and recall increased to 76.37% and 74.08%, respectively. The HD95 was reduced further to 12.48 pixels. Adding PEGB produced an additional improvement, yielding a Dice score of 76.10%, an IoU of 61.42%, and a HD95 of 11.73 pixels. This indicates that selectively controlling the contribution of the adaptive intensity representation improved both tumour-region overlap and boundary agreement. The addition of boundary supervision increased the Dice score to 76.95% and reduced the HD95 to 10.88 pixels. The improvement in boundary distance is consistent with the intended role of explicit contour supervision. Adding segmentation-error supervision further increased the Dice to 77.81% and the IoU to 63.68%, while reducing the HD95 to 10.07 pixels. Finally, the complete BCIW-Net, which additionally incorporates adaptive-window regularisation through $L_{\mathrm{window}}$, achieved the best overall performance, with a Dice score of 78.68%, an IoU of 64.85%, a precision of 79.87%, a recall of 77.52%, and a HD95 of 9.19 pixels. Relative to the V1 baseline, the complete BCIW-Net improved the Dice and IoU by 8.42 and 10.70 percentage points, respectively. Precision and recall increased by 8.44 and 8.40 percentage points, while the HD95 decreased from 15.84 to 9.19 pixels. Because the ablation is cumulative, these changes should be interpreted as order-dependent contributions under the evaluated sequence of model modifications.

#### 4.5.2. Effect of Input Image Resolution

The effect of input resolution was evaluated using the complete BCIW-Net architecture with identical optimisation settings across all resolutions. As shown in Table 7, increasing the input resolution from 128×128 to 256×256 progressively improved segmentation performance. The Dice score increased from 75.26% to 78.68%, while the IoU increased from 60.34% to 64.85%, corresponding to gains of 3.42 and 4.51 percentage points, respectively. Precision and recall also improved from 76.18% and 74.37% at 128×128 to 79.87% and 77.52% at 256×256. Over the same range, the HD95 decreased from 12.84 to 9.19 pixels, indicating improved agreement between the predicted and reference tumour boundaries as the spatial resolution increased. Increasing the input resolution beyond 256×256 did not produce further improvements. At 320×320, the Dice and IoU scores decreased slightly to 78.41% and 64.49%, respectively, while the HD95 increased to 9.42 pixels. At 384×384, the Dice and IoU decreased further to 78.05% and 64.01%, with the HD95 increasing to 9.71 pixels. A similar trend was observed for precision and recall. These results indicate that 256×256 provides an effective balance between spatial detail and segmentation accuracy for the present dataset. Higher resolutions increase computational requirements without improving either region-overlap performance or boundary agreement. Therefore, 256×256 was adopted as the input resolution for the final BCIW-Net configuration, as it achieved the best overall performance across the five-fold patient-level cross-validation experiments.

Table 8 evaluates the cumulative contribution of the auxiliary loss terms while keeping the BCIW-Net architecture unchanged. Using only the primary segmentation loss, the model achieved a Dice score of 76.10% and an IoU of 61.42%. Adding boundary supervision improved these scores to 76.95% and 62.53%, respectively, indicating that explicit contour supervision supports more accurate tumour delineation. The addition of segmentation-error supervision further increased the Dice score to 77.81% and the IoU to 63.68%, suggesting that learning the spatial distribution of prediction errors supports error-gated adaptive blending. Finally, incorporating adaptive-window parameter regularisation produced the complete BCIW-Net, which achieved the best performance, with a Dice score of 78.68%, an IoU of 64.85%, a precision of 79.87%, and a recall of 77.52%. Overall, the complete loss formulation improved the Dice and the IoU by 2.58 and 3.43 percentage points, respectively, compared with the segmentation-only objective. These cumulative results demonstrate that the auxiliary objectives provide complementary improvements under the evaluated training configuration.

#### 4.5.3. Computational Complexity and Capacity-Controlled Analysis

We additionally compared the computational requirements of BCIW-Net with those of the evaluated baseline architectures using the same input resolution and inference environment. As shown in Table 9, BCIW-Net contains 15.19 million trainable parameters and requires 53.11 GFLOPs per forward pass, with a mean inference time of 3.35±0.15 ms per slice. This computational footprint is lower than that of U-Net, Attention U-Net, UNet++, ResUNet, Swin-UNet, and Mamba-UNet in both parameter count and FLOPs, while remaining comparable in inference latency to the lighter architectures. EfficientUNet contains fewer parameters and achieves the lowest inference time, but requires more FLOPs than BCIW-Net. These results indicate that the improved segmentation performance of BCIW-Net is not simply explained by a larger model capacity or higher computational cost. To further isolate the contribution of the proposed intensity-adaptation mechanism, we performed a controlled windowing-specific ablation in which the network architecture, optimisation settings, loss formulation, data partitioning, and evaluation procedure were kept unchanged while only the windowing strategy was varied. The progressive improvement from fixed to globally learnable, spatially adaptive, and boundary-conditioned windowing provides additional evidence that the observed gains are associated with the proposed adaptive intensity mechanism rather than increased architectural capacity.

#### 4.5.4. Windowing-Specific Analysis

The cumulative ablation study examines the progressive construction of BCIW-Net by adding architectural components and loss terms in sequence. While this analysis shows how performance changes as the complete model is assembled, it does not isolate the effect of the intensity windowing mechanism itself. To address this, we conducted an additional windowing-specific ablation in which the network architecture, training protocol, loss formulation, data split, and evaluation procedure were kept unchanged, while only the windowing strategy was varied. Five configurations were evaluated. The first uses a fixed-intensity window with predefined parameters. The second replaces the fixed parameters with a globally learnable window optimized jointly with the segmentation network. The third introduces spatial adaptation, allowing the window parameters to vary across image locations. The fourth conditions the adaptive window on boundary information. The final configuration corresponds to the complete BCIW-Net, which combines boundary-conditioned adaptive windowing with the predicted-error-gated blending mechanism used in the proposed framework. This controlled setup allows the effect of increasingly adaptive windowing strategies to be examined independently of other architectural changes.

As shown in Table 10, segmentation performance improves progressively as the windowing strategy becomes more adaptive. The fixed-window configuration achieves a Dice score of 74.36% and an IoU of 59.18%, with a HD95 of 14.82 pixels. Replacing the predefined window with a globally learnable formulation increases the Dice to 75.41% and the IoU to 60.53%, while reducing the HD95 to 13.71 pixels. This indicates that optimizing the window parameters directly from the training data provides an advantage over using a fixed-intensity transformation. Introducing spatially adaptive windowing yields a further improvement, with the Dice increasing to 76.52% and the HD95 decreasing to 12.46 pixels. This suggests that a single global transformation does not fully account for regional intensity variations within the same slice. Allowing the window parameters to vary spatially gives the model greater flexibility to adjust the representation according to local image characteristics. Conditioning the adaptive window on boundary information improves the Dice to 77.63% and the IoU to 63.44%, while the HD95 decreases to 10.93 pixels. The reduction in HD95 value is consistent with the intended role of boundary conditioning, since the window generator receives information about regions near lesion margins where the contrast between the target and surrounding tissues may be limited. The full BCIW-Net achieves the best result across all reported metrics, with a Dice score of 78.68%, an IoU of 64.85%, a precision of 79.87%, a recall of 77.52%, and a HD95 of 9.19 pixels. Relative to the fixed-window configuration, the complete model improves the Dice by 4.32 percentage points and the IoU by 5.67 percentage points, while reducing the HD95 by 5.63 pixels. Compared with boundary-conditioned adaptive windowing alone, the complete BCIW-Net provides a further gain of 1.05 percentage points in the Dice and reduces the HD95 by 1.74 pixels. This analysis separates the contribution of the proposed windowing mechanism from the cumulative architectural changes considered in the main ablation study. The progression from fixed, globally learnable, and spatially adaptive windowing to boundary-conditioned adaptation shows a consistent improvement in both overlap-based and boundary-based measures, while the full BCIW-Net provides the strongest overall performance.

### 4.6. Qualitative Segmentation Results

**Figure 5:**
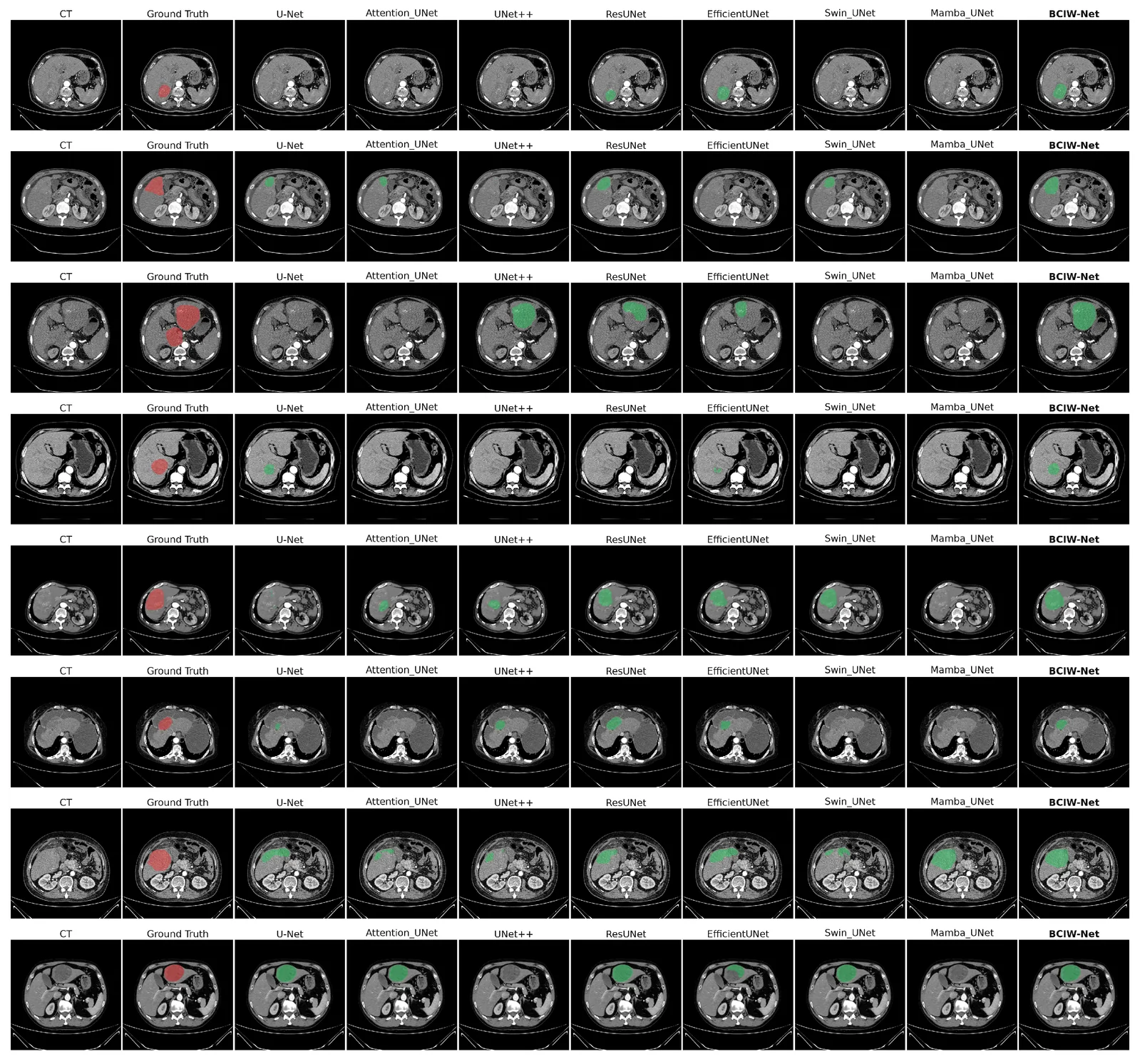
Qualitative comparison of HCC tumour segmentation on representative CT slices from the independent test set. From left to right: the original CT image, ground-truth annotation, and predictions generated by U-Net, Attention U-Net, UNet++, ResUNet, EfficientUNet, Swin-UNet, Mamba-UNet, and the proposed BCIW-Net. Ground-truth and predicted tumour regions are shown in red and green, respectively. In these examples, BCIW-Net demonstrates closer spatial agreement with the ground-truth annotations, including more accurate tumour localisation, better preservation of lesion morphology, and clearer boundary delineation, particularly for tumours with heterogeneous enhancement and poorly defined margins.

**Figure 6:**
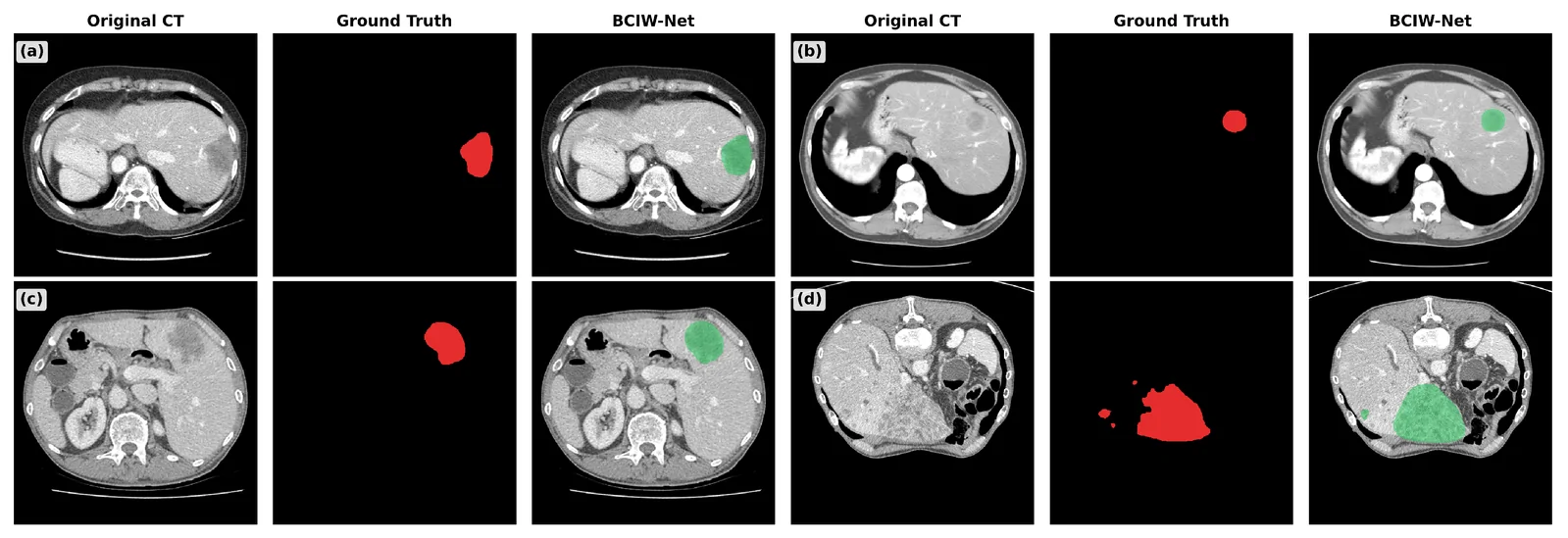
Representative tumour segmentation results produced by BCIW-Net on the LiTS2017 dataset. (**a**) Segmentation of a relatively small tumour located near the peripheral region of the liver. (**b**) Detection of a small, approximately rounded tumour within the liver parenchyma. (**c**) Segmentation of a tumour with a relatively compact morphology in a different anatomical region. (**d**) Segmentation of a larger, irregularly shaped tumour exhibiting a more complex spatial extent. For each subfigure, the original CECT image, the corresponding ground-truth tumour annotation in red, and the segmentation predicted by BCIW-Net in green are shown from left to right. The selected cases shows variations in tumour size, morphology, anatomical location, and imaging appearance. BCIW-Net successfully identifies both small and large tumours, with predicted masks showing good spatial correspondence with the expert annotations.

Figure 5 compares BCIW-Net with seven representative segmentation architectures on selected PLC-CECT cases. In the examples shown, BCIW-Net produces masks that show closer agreement with the ground-truth annotations, particularly for small, low-contrast, and morphologically irregular tumours. Several competing methods exhibit incomplete tumour coverage or inaccurate boundary delineation, whereas BCIW-Net generally preserves tumour extent and irregular boundary details more effectively. These observations are consistent with the quantitative results reported in Table 3. To further examine performance on an independent dataset, Figure 6 presents representative tumour segmentation results produced by BCIW-Net on the LiTS2017 dataset. Each example shows the original contrast-enhanced CT image, the corresponding ground-truth tumour annotation, and the predicted segmentation. The selected cases include tumours with different sizes, morphologies, anatomical locations, and imaging appearances, showing the heterogeneity present in the LiTS2017 dataset. Across these examples, the segmentations produced by BCIW-Net generally agree closely with the expert annotations. In the first two cases, the proposed method accurately delineates relatively small lesions while preserving their shape and spatial location. The third example demonstrates accurate localisation and segmentation of a moderately sized tumour with a heterogeneous appearance. In the fourth example, BCIW-Net captures the overall extent of a large and morphologically complex lesion, although small differences between the predicted and ground-truth boundaries are visible along some peripheral regions. Despite these challenging characteristics, the predicted masks preserve the main morphological features and spatial extent of the tumours.

#### 4.6.1. Interpretability Analysis of BCIW-Net

**Figure 7:**
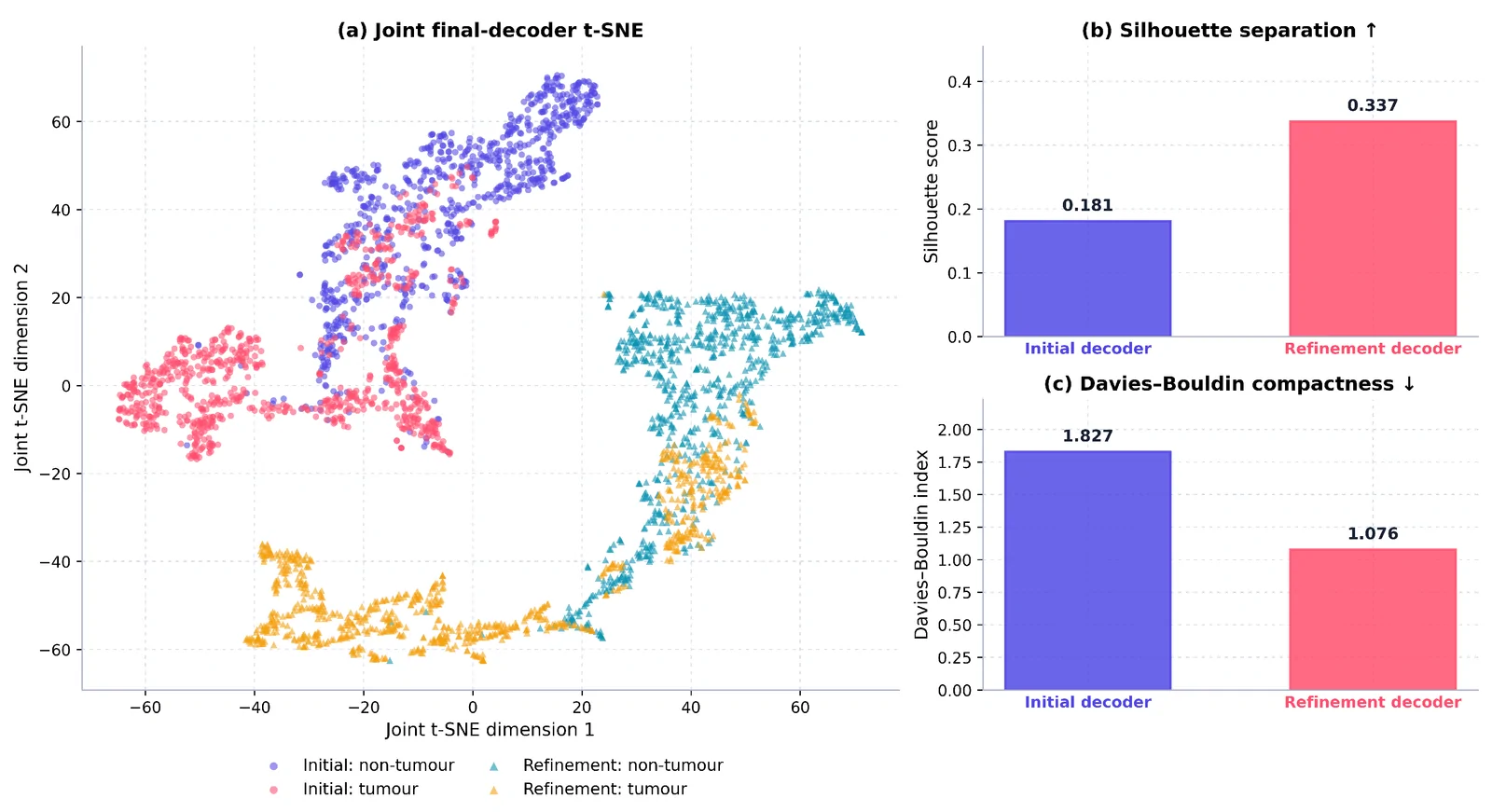
Analysis of tumour and non-tumour feature separability in the BCIW-Net decoders. (**a**) Joint t-SNE projection of pixel-wise features extracted from the initial and refinement decoders. Non-tumour and tumour features from the initial decoder are shown as blue and pink circles, respectively, while those from the refinement decoder are shown as cyan and orange triangles. The refinement features form more compact class-specific groups with less visual overlap between tumour and non-tumour samples. (**b**) Silhouette scores calculated from the PCA-reduced features of the initial and refinement decoders. Higher values indicate greater inter-class separation and intra-class cohesion. The score increases from 0.181 to 0.337. (**c**) Davies–Bouldin indices calculated from the PCA-reduced features, where lower values indicate more compact and well-separated clusters. The index decreases from 1.827 to 1.076. Together, the visualisation and clustering metrics indicate that the refinement stage produces a more discriminative feature representation.

Figure 7 presents a combined qualitative and quantitative evaluation of the feature representations generated by the initial and refinement decoders of BCIW-Net. From each of 14 HCC CT slices, 60 tumour and 60 non-tumour locations were sampled, resulting in 1680 balanced 96-dimensional feature vectors for each decoder. To obtain a directly comparable visualisation, the features from both decoders were jointly standardised, reduced to 32 principal components using principal component analysis (PCA), and projected into a common 2D space using a single t-SNE model. The t-SNE parameters included a perplexity of 35, PCA initialisation, an automatically selected learning rate, 1800 iterations, and a fixed random seed of 42. The joint embedding shown in panel (a) enables direct visual comparison of the two feature representations within the same coordinate system, revealing clear reorganisation of the tumour and non-tumour features following refinement. To quantify class separability in the higher-dimensional feature space, the features from each decoder were standardised and reduced separately to 32 principal components before the Silhouette score and Davies–Bouldin index were calculated. Importantly, these metrics were calculated from the PCA-reduced features rather than the 2D t-SNE projections. As shown in panel (b), the Silhouette score increased from 0.181 to 0.337, indicating improved separation between tumour and non-tumour features. Panel (c) shows that the Davies–Bouldin index decreased from 1.827 to 1.076, indicating greater within-class compactness and between-class separation. Together, the qualitative visualisation and quantitative metrics provide evidence that the refinement decoder generates a more structured and discriminative feature representation for HCC tumour segmentation.

**Figure 8:**
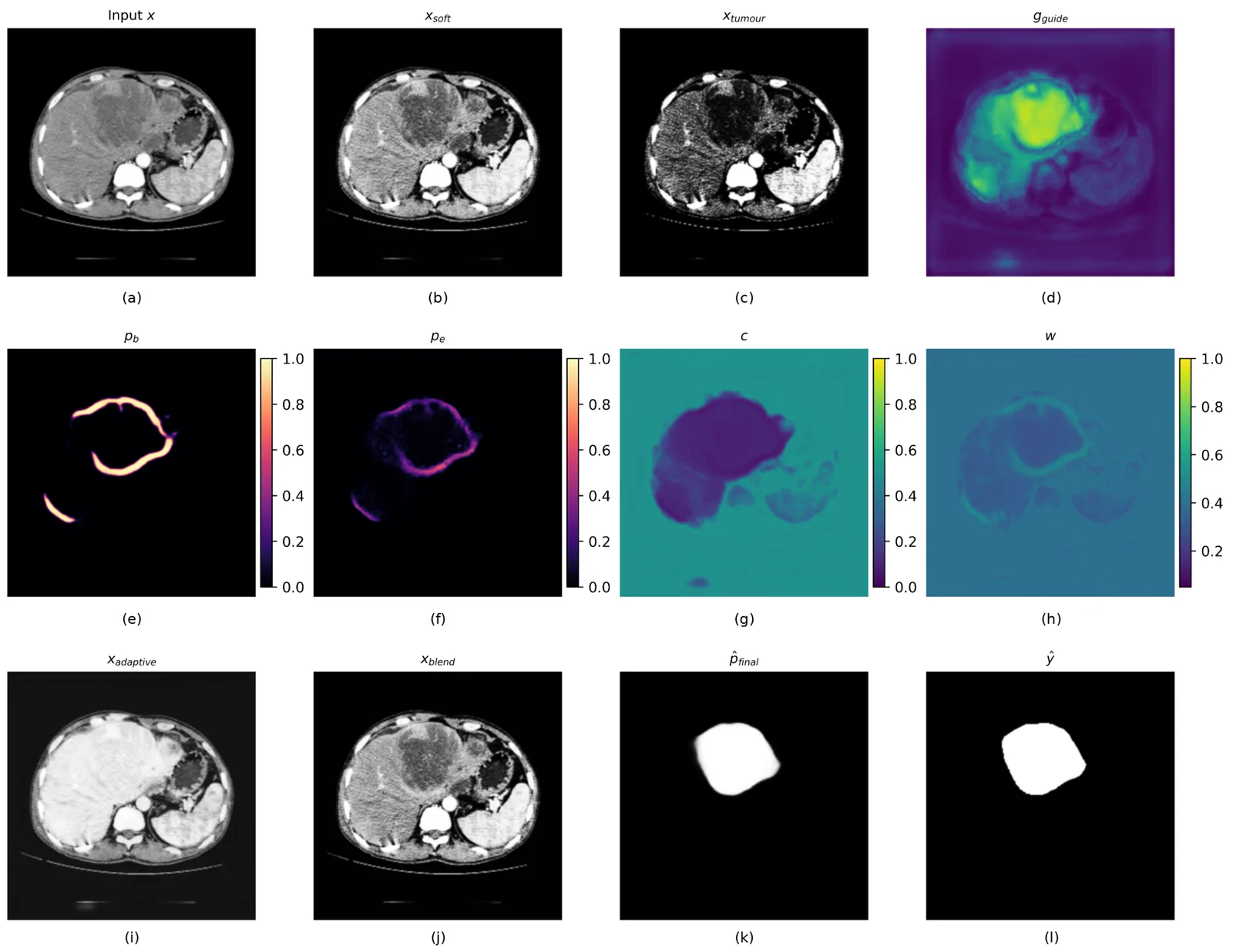
Representative intermediate representations generated by BCIW-Net during HCC segmentation in CECT images. (**a**) Original normalised CT image. (**b**) Soft-tissue intensity representation generated using the fixed soft-tissue window transformation. (**c**) Tumour-focused intensity representation generated using the fixed tumour-focused window transformation. (**d**) Latent guidance activation produced by the first-stage encoder–decoder, showing the approximate tumour location. (**e**) Predicted boundary probability map highlighting the tumour contour. (**f**) Predicted error-proxy map highlighting regions expected to be difficult to segment. (**g**) Spatially adaptive window-centre map predicted by the BCWG. (**h**) Spatially adaptive window-width map predicted by the BCWG. (**i**) Adaptive intensity representation generated from the predicted centre and width maps. (**j**) Error-gated blended representation obtained by weighting the adaptive and soft-tissue representations according to the predicted error-proxy map. (**k**) Final tumour probability map generated by the refinement network. (**l**) Final binary tumour mask obtained by thresholding the probability map. These sequential visualisations show how BCIW-Net combines boundary information, predicted segmentation difficulty, and adaptive intensity windowing during tumour delineation, while providing intermediate representations for qualitative inspection.

Figure 8 presents representative intermediate feature representations generated by BCIW-Net during inference, showing how the boundary-conditioned adaptive-windowing mechanism transforms the input image to support tumour segmentation. The original normalised CT image in panel (a) is first processed using two complementary differentiable sigmoid-based intensity transformations. These transformations produce a soft-tissue representation in panel (b), which preserves broad anatomical information, and a tumour-focused representation in panel (c), which enhances tumour-related intensity contrast. The two fixed representations are then processed by the initial encoder–decoder to generate latent guidance activation in panel (d), which indicates the approximate location of the tumour, together with a boundary probability map in panel (e) and an error-proxy map in panel (f). The boundary probability map shows strong responses around the tumour contour, whereas the error-proxy map highlights regions in which the initial segmentation is predicted to be less reliable.

**Figure 9:**
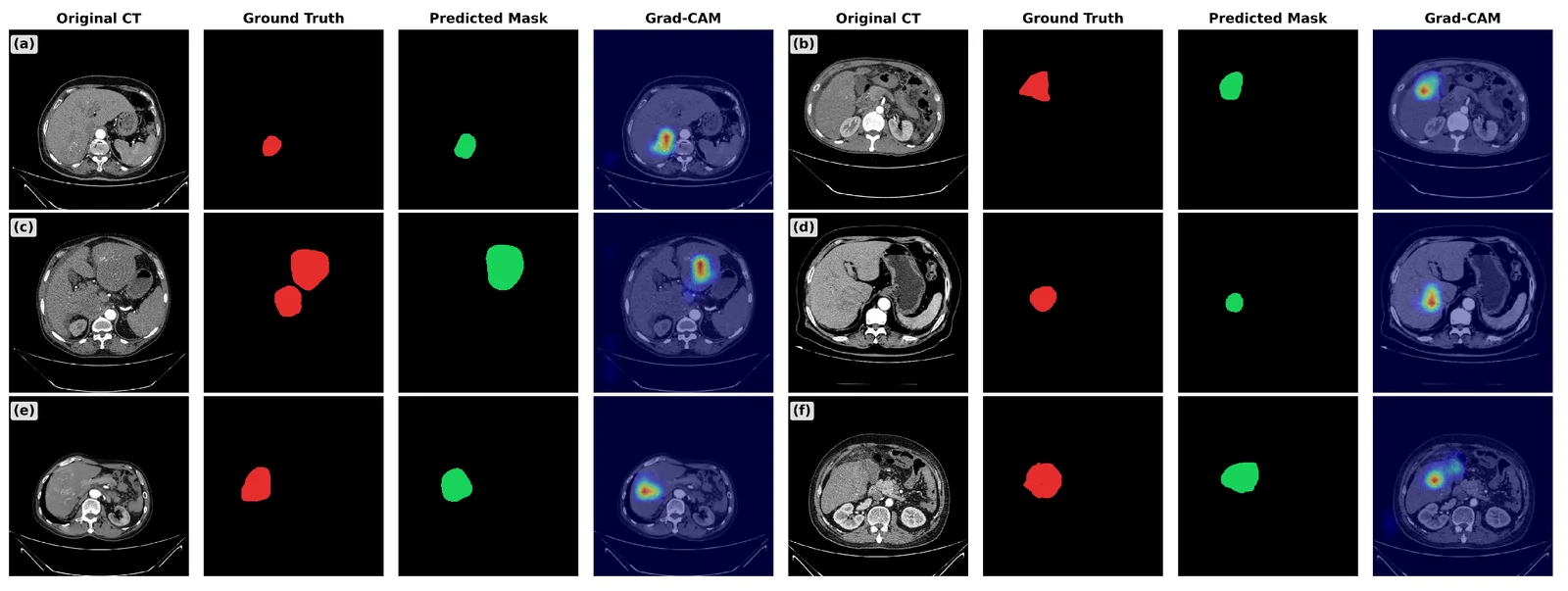
Grad-CAM visualisation of BCIW-Net for six representative HCC segmentation cases. (**a**) Localised tumour region with concentrated Grad-CAM activation; (**b**) small tumour region exhibiting a spatially focused activation response; (**c**) multiple tumour regions with activation concentrated around the corresponding lesions; (**d**) tumour region showing localised activation within the liver; (**e**) relatively small lesion with a distinct activation hotspot; and (**f**) tumour region exhibiting a confined activation pattern. Each subfigure presents the original contrast-enhanced CT image, the corresponding expert reference tumour annotation (red), the tumour segmentation predicted by BCIW-Net (green), and the Grad-CAM activation map generated from the final segmentation network. Warmer colours indicate stronger activation, whereas cooler colours represent lower activation. Across the representative cases, the activation maps generally correspond to the annotated tumour regions, suggesting that BCIW-Net responds to tumour-related image features during segmentation. These visualisations provide qualitative interpretability and should be considered alongside the quantitative evaluation.

The BCWG uses these intermediate representations to estimate spatially varying window centre and width maps, shown in panels (g) and (h), respectively. Unlike conventional CT windowing, which applies a single global window to the entire image, the predicted parameter maps vary according to local anatomical and morphological characteristics. This spatial variation allows the intensity transformation to adapt to different image regions. Applying the estimated parameters produces the adaptive intensity representation in panel (i), in which tumour regions are selectively enhanced. This representation is subsequently combined with the fixed soft-tissue representation through an error-gated blending mechanism to produce the blended image in panel (j). The predicted error-proxy map controls the contribution of adaptive enhancement, increasing it in potentially challenging regions while preserving stable anatomical information elsewhere. Finally, the refinement network generates the tumour probability map in panel (k), which is converted into the binary segmentation mask in panel (l) using the selected decision threshold. Collectively, these visualisations provide qualitative insight into the intermediate processes of tumour localisation, boundary estimation, adaptive intensity transformation, error-gated blending, and segmentation refinement. To further examine the spatial focus of the learned model, Figure 9 presents Grad-CAM visualisations for representative HCC cases processed by BCIW-Net. Each example shows the original CT image, expert annotation, predicted tumour mask, and corresponding Grad-CAM activation map. The Grad-CAM maps were generated from the final segmentation network to identify the image regions most strongly associated with the predicted tumour segmentation.

Across the representative cases, the strongest activation responses are concentrated within or near the tumour regions identified by the expert annotations. For relatively small lesions, shown in panels (a), (b), (d), and (e), the activation maps contain compact high-response regions centred on the tumours. This pattern suggests that tumour-related image features contribute strongly to the corresponding predictions. For the larger or more heterogeneous lesions in panels (c) and (f), the activation covers a broader area of the tumour while remaining largely confined to the pathological region. Relatively limited activation is observed in the surrounding liver parenchyma and adjacent abdominal structures, suggesting that the network assigns greater importance to tumour-associated regions than to the surrounding anatomy. The spatial correspondence between the Grad-CAM responses, predicted masks, and expert annotations suggests that the features used by BCIW-Net are spatially associated with clinically relevant tumour regions. However, Grad-CAM does not provide a causal explanation of model behaviour. These visualisations should therefore be interpreted as qualitative supporting evidence that complements, rather than validates, the quantitative segmentation results.

#### 4.6.2. Predicted Error Map Validation

**Figure 10:**
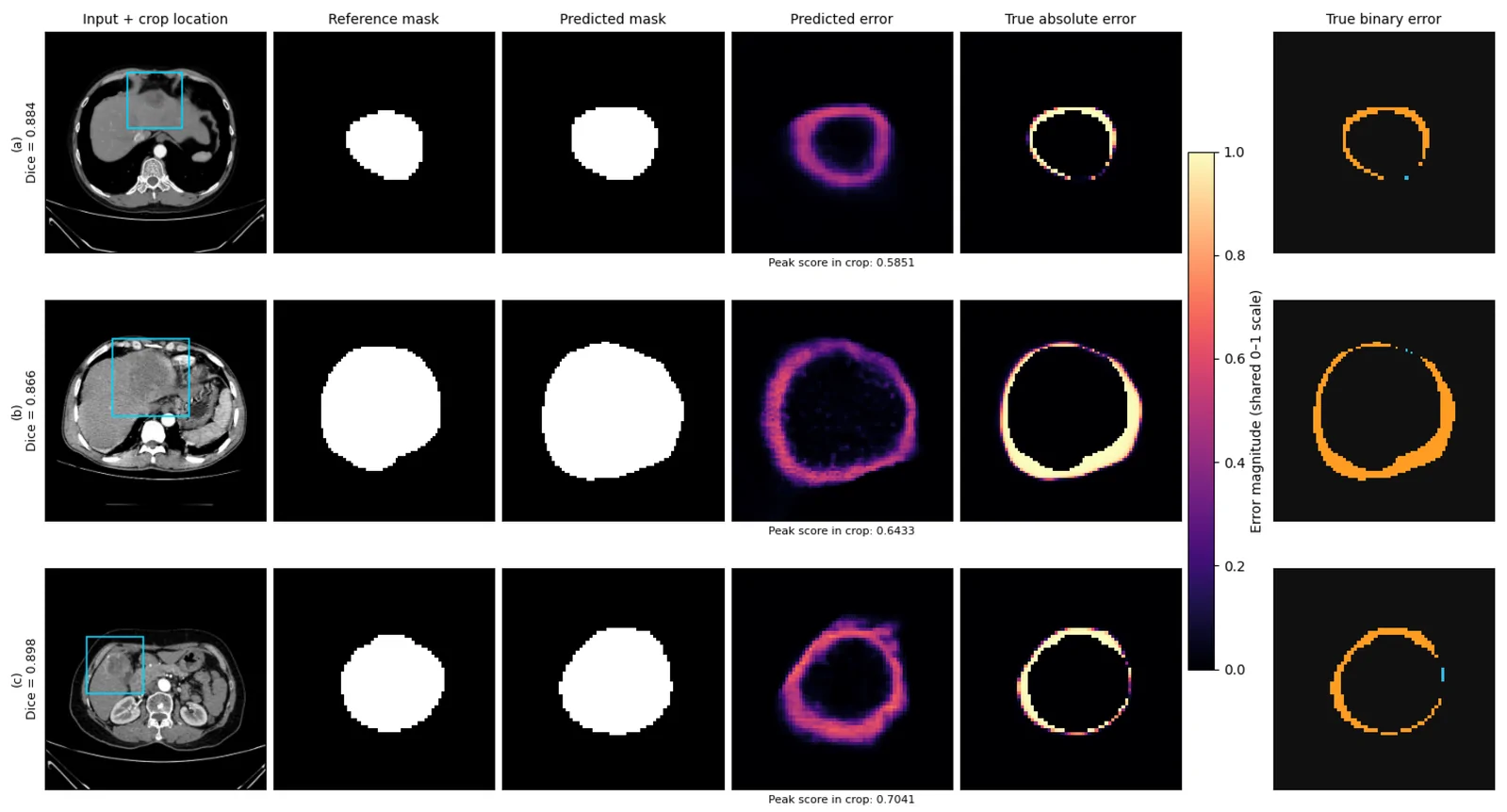
Qualitative validation of the predicted error-proxy map for three representative HCC segmentation cases. (**a**) A case with a Dice score of 0.884, showing localised segmentation discrepancies around the tumour boundary; (**b**) a case with a Dice score of 0.866, exhibiting more extensive boundary disagreement and stronger predicted-error responses; and (**c**) a case with a Dice score of 0.898, demonstrating relatively close agreement between the predicted and reference masks, with residual errors concentrated along the tumour margin. The cyan rectangular boxes in the original CECT images indicate the selected tumour regions of interest used for the corresponding cropped visualisations. Each row presents the original CT image with the selected crop location, reference tumour mask, predicted segmentation mask, predicted error-proxy map $p_{e}$, true absolute error, and binary segmentation-error map. The true absolute error represents the pixel-wise discrepancy between the final segmentation probability and the reference annotation, whereas the binary error map identifies disagreement between the thresholded prediction and the reference mask. Orange pixels indicate false-positive regions, while cyan pixels indicate false-negative regions. Across the three cases, higher predicted-error responses are predominantly concentrated around tumour boundaries and spatially correspond to regions of actual segmentation disagreement. The broader, continuous response of $p_{e}$ reflects its role as a proxy for segmentation difficulty rather than a binary error indicator.

To examine whether the predicted error map $p_{e}$ corresponds to regions of actual segmentation error, we performed a qualitative comparison between the predicted error response and error maps derived from the final segmentation output. Figure 10 presents representative cases showing the input CT image, reference tumour mask, predicted segmentation mask, predicted error map, true absolute error, and binary segmentation-error map. Across the selected examples, elevated responses in $p_{e}$ are concentrated predominantly around tumour boundaries, where disagreement between the predicted and reference masks occurs. This spatial correspondence is particularly evident in regions associated with false-positive and false-negative boundary discrepancies. The predicted error map also shows a broader response around uncertain tumour margins than the binary error map, which is consistent with its role as a continuous proxy for segmentation difficulty rather than a binary indicator of misclassified pixels. These observations provide qualitative evidence that $p_{e}$ captures spatial patterns associated with segmentation error and support its use as the gating signal in the predicted-error-gated blending mechanism. The error map should nevertheless be interpreted as an error proxy rather than as a calibrated estimate of epistemic or aleatoric uncertainty.

## 5. Discussion

The proposed BCIW-Net integrates a differentiable and spatially adaptive intensity transformation within an end-to-end framework for HCC segmentation in contrast-enhanced CT. On the PLC-CECT cohort, the method achieved a mean Dice score of 78.68% and an IoU of 64.85% under five-fold patient-level cross-validation, with improved overlap and boundary-localisation performance relative to the evaluated CNN-, Transformer-, and state-space-based models. The windowing-specific ablation further showed progressive gains when moving from fixed-intensity transformation to globally learnable, spatially adaptive, and boundary-conditioned formulations, supporting the contribution of adaptive intensity modelling to the observed performance. Because the comparative analysis included seven representative segmentation architectures, these gains should be interpreted relative to the evaluated baselines rather than as evidence of superiority over all contemporary methods. The proposed mechanism is clinically motivated by the practice of adjusting image contrast during CT interpretation; however, the learned parameters *c* and *w* operate in normalised-intensity space and parameterise a sigmoid transformation. Therefore, they are not numerically equivalent to conventional radiological window level and width, and should not be interpreted as a direct simulation of radiologist-controlled CT windowing. Several limitations should be considered when interpreting these findings. The primary analysis included 94 patients with pathologically confirmed HCC, and although patient-level cross-validation was used, validation in larger independent multicentre cohorts remains necessary. The LiTS2017 analysis provides cross-dataset evidence because no LiTS data were used for training, fine-tuning, validation, checkpoint selection, or threshold optimisation; however, the publicly labelled training cohort of the original challenge was used, and the results should therefore be regarded as preliminary external evaluation rather than definitive clinical validation. The present study also did not separately assess several clinically challenging scenarios, including small or low-contrast lesions, infiltrative or poorly marginated tumours, lesions adjacent to hepatic vessels or bile ducts, post-treatment appearances, heterogeneous or necrotic lesions, and images affected by motion, beam-hardening, or other artifacts. In addition, variation in scanner vendor, reconstruction settings, slice thickness, contrast timing, and acquisition protocol may influence model performance. Further evaluation on larger multicentre datasets, diverse acquisition conditions, post-treatment imaging, and multiphasic or volumetric CT is therefore warranted.

## 6. Conclusions

We introduced BCIW-Net, a boundary- and error-conditioned adaptive intensity framework for HCC segmentation in CECT. By integrating spatially adaptive intensity transformation with boundary guidance, error-aware blending, and two-stage refinement, the proposed approach achieved higher segmentation and boundary-localisation performance than the evaluated comparison architectures on the PLC-CECT cohort. The LiTS2017 experiment further provided encouraging evidence of cross-dataset generalisation without dataset-specific fine-tuning. These findings support the use of adaptive intensity modelling as a complementary strategy for improving tumour representation and delineation in challenging CT images. Further evaluation on larger multicentre cohorts, diverse acquisition protocols, post-treatment cases, and prospective clinical datasets is required to establish the robustness, generalisability, and potential clinical applicability of BCIW-Net.

## Figures and Tables

**Table 1:** Clinical imaging challenges relevant to HCC segmentation and corresponding computational considerations.

Imaging Challenge	Effect on Segmentation	Computational Consideration	Relation to BCIW-Net
Variation across contrast phases	Tumour-to-liver contrast and lesion conspicuity differ between arterial, portal venous, and delayed acquisitions	Phase-specific modelling or multiphase feature fusion	The present implementation uses single-phase CECT; multiphase modelling remains a future extension
Variable LI-RADS imaging appearance	Differences in enhancement, morphology, and margin visibility can complicate lesion delineation	Context-aware and boundary-aware feature modelling	Boundary conditioning may assist tumour delineation, although BCIW-Net does not perform LI-RADS categorisation
Post-treatment imaging changes	Necrosis, perfusional alterations, treatment cavities, and persistent enhancement may obscure residual viable tumour	Treatment-specific or longitudinal modelling	Post-treatment appearances were not separately evaluated in the present study
Poorly defined, infiltrative, or low-contrast tumours	Weak tumour–parenchyma contrast increases the risk of incomplete or excessive segmentation	Adaptive intensity representation and explicit boundary supervision	Addressed through spatially adaptive intensity transformation and boundary conditioning
Tumours adjacent to vessels or bile ducts	Similar enhancement and close anatomical relationships may lead to inaccurate tumour extension	Anatomical context, boundary supervision, and refinement	Boundary- and error-aware guidance may reduce local segmentation ambiguity
Limited availability of contrast-enhanced CT	Absence of enhancement cues can reduce tumour conspicuity	Non-contrast-specific enhancement, structural priors, or frequency-domain approaches	BCIW-Net has not been validated on non-contrast CT

**Table 2:** Five-fold patient-level cross-validation performance of the proposed BCIW-Net on the PLC-CECT dataset. The best result in each column is highlighted in bold.

Fold	Dice (%)	IoU (%)	Precision (%)	Recall (%)	Specificity (%)	HD95 (Pixels)
Fold 1	78.42	64.50	79.61	77.27	97.76	9.24
Fold 2	78.71	64.89	79.90	77.55	97.83	8.97
Fold 3	77.34	63.05	78.51	76.20	97.73	10.31
Fold 4	80.26	67.03	81.47	79.08	97.89	8.35
Fold 5	78.67	64.84	79.86	77.52	97.84	9.06
**Mean ± SD**	**78.68 ± 1.05**	**64.86 ± 1.42**	**79.87 ± 1.07**	**77.52 ± 1.03**	**97.81 ± 0.06**	**9.19 ± 0.71**

**Table 3:** Quantitative comparison of BCIW-Net with representative convolutional, Transformer-based, and state-space segmentation architectures on the PLC-CECT dataset using five-fold patient-level cross-validation. The best result in each column is highlighted in bold.

Method	Dice (%)	IoU (%)	Precision (%)	Recall (%)	Specificity (%)	HD95 (Pixels)
U-Net [10]	70.08	53.94	71.26	68.94	96.31	14.82
Attention U-Net [13]	72.20	56.49	73.05	71.37	96.74	13.74
UNet++ [14]	73.99	58.71	75.18	72.83	97.02	12.96
ResUNet [15]	75.51	60.66	76.44	74.61	97.18	11.85
EfficientUNet [38]	75.13	60.17	76.93	73.42	97.09	12.14
Swin-UNet [39]	76.71	62.23	77.61	75.84	97.43	10.87
Mamba-UNet [40]	77.19	62.85	78.23	76.17	97.56	10.12
**Proposed (BCIW-Net)**	**78.68**	**64.85**	**79.87**	**77.52**	**97.81**	**9.19**

**Table 4:** Tumour-positive-slice and boundary-sensitive evaluation of Mamba-UNet and the proposed BCIW-Net on the PLC-CECT test set. The test set contained 610 tumour-positive slices. The best result in each metric column is highlighted in bold.

Method	Tumour-Positive-Slice Dice (%)	HD95 (Pixels)	Missed Tumour Slices	False-Positive Slices
Mamba-UNet	74.86	11.42	21/610 (3.44%)	26
**BCIW-Net**	**76.73**	**9.87**	**14/610 (2.30%)**	**19**

**Table 5:** Cross-study comparison of published liver tumour segmentation methods evaluated on the LiTS2017 dataset. The highest reported value is highlighted in bold.

Method	Year	Type	Tumour Dice (%)
MA-cGAN [41]	2024	Hybrid	78.53
RHEU-Net [42]	2024	Hybrid	70.19
ELANRes-MSCA-UNet [43]	2025	Hybrid	72.90
Improved Swin-UNet [44]	2025	Transformer	76.14
G-UNETR++ [45]	2026	Hybrid	84.40
**BCIW-Net**	2026	Hybrid	**89.84**

**Table 6:** Cumulative ablation study of BCIW-Net on the PLC-CECT dataset using five-fold patient-level cross-validation. FITs denotes fixed sigmoid-based intensity transformations, 2S denotes the two-stage architecture, BCWG denotes the Boundary-Conditioned Window Generator, and PEGB denotes the predicted-error-gated blending mechanism. Results are reported as the mean across the five test folds. The best result in each metric column is highlighted in bold.

Model Variant	Components							Performance				
	**FITs**	**2S**	**BCWG**	**PEGB**	$L_{\mathrm{boundary}}$	$L_{\mathrm{error}}$	$L_{\mathrm{window}}$	**Dice (%)**	**IoU (%)**	**Precision (%)**	**Recall (%)**	**HD95 (Pixels)**
V1: Single-stage raw-input baseline	×	×	×	×	×	×	×	70.26	54.15	71.43	69.12	15.84
V2: V1 (+) FIT	✓	×	×	×	×	×	×	72.62	57.01	73.74	71.53	14.72
V3: V2 (+) 2S	✓	✓	×	×	×	×	×	73.91	58.62	74.98	72.87	13.61
V4: V3 (+) BCWG	✓	✓	✓	×	×	×	×	75.21	60.27	76.37	74.08	12.48
V5: V4 (+) PEGB	✓	✓	✓	✓	×	×	×	76.10	61.42	77.04	75.18	11.73
V6: V5 (+$L_{\mathrm{boundary}}$)	✓	✓	✓	✓	✓	×	×	76.95	62.53	77.89	76.03	10.88
V7: V6 (+$L_{\mathrm{error}}$)	✓	✓	✓	✓	✓	✓	×	77.81	63.68	78.91	76.74	10.07
V8: Full BCIW-Net	✓	✓	✓	✓	✓	✓	✓	**78.68**	**64.85**	**79.87**	**77.52**	**9.19**

**Table 7:** Segmentation performance of BCIW-Net at different input resolutions on the PLC-CECT dataset using five-fold patient-level cross-validation. All configurations were trained using identical optimisation settings. The best result for each metric is highlighted in bold.

Input Resolution	Dice (%)	IoU (%)	Precision (%)	Recall (%)	HD95 (Pixels)
128×128	75.26	60.34	76.18	74.37	12.84
192×192	77.05	62.67	78.01	76.11	10.63
256×256	**78.68**	**64.85**	**79.87**	**77.52**	**9.19**
320×320	78.41	64.49	79.54	77.31	9.42
384×384	78.05	64.01	79.16	76.98	9.71

**Table 8:** Cumulative loss-function ablation of BCIW-Net on the PLC-CECT dataset using five-fold patient-level cross-validation. All variants use the same architecture, including fixed-intensity transformations, the two-stage encoder–decoder, BCWG, and predicted-error-gated blending. Starting from the segmentation-only objective, auxiliary loss terms are added cumulatively. The best result in each column is highlighted in bold.

Variant	$L_{\mathrm{boundary}}$	$L_{\mathrm{error}}$	$L_{\mathrm{window}}$	Dice (%)	IoU (%)	Precision (%)	Recall (%)
Segmentation loss only	×	×	×	76.10	61.42	77.04	75.18
$+L_{\mathrm{boundary}}$	✓	×	×	76.95	62.53	77.89	76.03
$+L_{\mathrm{error}}$	✓	✓	×	77.81	63.68	78.91	76.74
BCIW-Net: $+L_{\mathrm{window}}$	✓	✓	✓	**78.68**	**64.85**	**79.87**	**77.52**

**Table 9:** Computational complexity and inference efficiency of BCIW-Net and the evaluated baseline architectures. GFLOPs are reported for a single forward pass. Inference time is reported as mean ± standard deviation per slice.

Model	Parameters (M)	GFLOPs	Inference Time (ms/Slice)
U-Net	30.34	101.56	3.63±0.14
Attention U-Net	30.86	104.79	4.39±0.20
UNet++	35.17	255.11	8.28±0.28
ResUNet	30.34	101.56	3.93±0.11
EfficientUNet	11.51	67.77	2.58±0.17
Swin-UNet	35.92	111.16	4.42±0.26
Mamba-UNet	37.33	112.37	4.58±0.21
BCIW-Net	15.19	53.11	3.35±0.15

**Table 10:** Windowing-specific ablation study using five-fold patient-level cross-validation on the PLC-CECT cohort. All configurations use the same network architecture, training protocol, loss formulation, data partitioning, and evaluation procedure; only the windowing strategy is varied. The best result for each metric is highlighted in bold.

Windowing Strategy	Dice (%)	IoU (%)	Precision (%)	Recall (%)	HD95 (Pixels)
Fixed-intensity window	74.36	59.18	75.42	73.33	14.82
Learnable global window	75.41	60.53	76.31	74.53	13.71
Spatially adaptive window	76.52	61.97	77.38	75.68	12.46
Boundary-conditioned adaptive window	77.63	63.44	78.69	76.60	10.93
Full BCIW-Net	**78.68**	**64.85**	**79.87**	**77.52**	**9.19**

## Data Availability

The datasets used in this study are publicly available. The PLC-CECT dataset and the LiTS2017 dataset can be accessed from their respective public repositories. No new datasets were generated as part of this study.
